# Similarities in the consumption trajectory of antibacterial drugs in the outpatient care sector in Germany from 1986 to 2022: identification of shared patterns, correlation analysis of prescribed defined daily dose and assessment of underlying influences

**DOI:** 10.1007/s00210-025-04165-0

**Published:** 2025-04-23

**Authors:** Lilly Josephine Bindel, Roland Seifert

**Affiliations:** Institute of Pharmacology, Hannover Medical School, 30625 Hannover, Germany

**Keywords:** Antimicrobial consumption, Antibiotic, Antimicrobial prescription, Antibiotic prescription, Germany, Arzneiverordnungsreport, Surveillance, Antibiotic stewardship, Correlation, Socioeconomic factors

## Abstract

Antibacterial drugs are widely used in modern medicine, but their efficacy is increasingly threatened by antimicrobial resistance and ongoing supply shortages. This study examines the relationships among the 15 most prescribed antibacterial substances in Germany, analysing the correlations and trends of prescribed defined daily dose (DDD) of antibacterial drugs within the outpatient care sector under statutory health insurance from 1986 to 2022. Three groups of drugs with similar consumption trajectories were identified. The first group, including doxycycline, phenoxymethylpenicillin and sulfamethoxazole-trimethoprim, showed declining trends with stable trajectories, its similarities being confirmed by overlapping indications such as respiratory tract infections and declining usage due to guideline changes. The second group, comprising amoxicillin-clavulanic acid, cefpodoxime and pivmecillinam, displayed increasing trends with continuous trajectories. The third group, including ciprofloxacin, clarithromycin and cefaclor, shared declining trends with fluctuating trajectories, often related to bacterial resistance rates and substitution by other antibacterial drugs. Frequently prescribed substances often show many and strong correlations in consumption, indicating that they are more related with other substances and being susceptible to external factors, such as changes in costs, sudden events like the COVID pandemic or guideline recommendations. Less commonly used medicines, typically for niche indications, tend to have a more stable consumption trend and being less influenced by broader trends, resulting in fewer significant correlations. Distinguishing direct effects of changes in consumption from one drug to another from common but unrelated responses to external events remains a challenge, as there are many factors that can influence consumption. The findings provide a better understanding of the interdependencies in the consumption of the most commonly prescribed antibacterial drugs, improving the understanding of potential influencing factors to support strategies for rational prescribing and antimicrobial stewardship.

**Supplementary Information:**

The online version contains supplementary material available at 10.1007/s00210-025-04165-0.

## Introduction

Antibacterial drugs are essential and frequently used in modern medicine (Cook and Wright 2022; Hutchings et al. 2019). Alarmingly, several problematic developments threaten their future effectiveness, such as increasing antimicrobial resistance (AMR) (UN 2024; IHME 2024; Salam et al. 2023) and persistent supply shortages (BfArM 2023; PharmNet.Bund 2025; SZ 2024; Berndt 2024).

Prescribing patterns of antibacterial drugs are complex and influenced by several factors. Previous research has shown that decreasing defined daily dose (DDD) costs encourage increases in consumption (Bindel and Seifert 2024a, 2024b), while increased use leads to growing bacterial resistance to the antibacterial drug in question (Bindel and Seifert 2024c, 2024d; Abejew et al. 2024; Ahmed et al. 2024). Furthermore, there are several other drivers that can influence physicians’ prescribing behaviour (Bindel and Seifert 2024a, 2024c; Rodrigues et al. 2013; Kasse et al. 2024; Merlo et al. 2025; van der Zande et al. 2019). However, the extent to which prescribing patterns are influenced is not exactly determinable.

The aim of this study is to analyse the consumption volumes and trends of antibacterial substances over a long period of time. A particular focus is the analysis of relationships between consumption trends of different antibacterial drugs. For this purpose, consumption data in terms of DDD prescriptions were analysed for the outpatient sector in Germany over the last decades. In addition, a correlation analysis was performed for the consumption of each of the 15 most commonly prescribed antibacterial drugs in 2022. Characteristics of the substances that may be responsible for their consumption trajectories and interdependencies with the consumption trends of other antibacterials are examined. Besides this, a hypothesis is that the more frequently a drug is prescribed, the more influence it has on less prescribed drugs. This would be reflected in a larger number of significant correlations, as there could be strong influences of dependent behaviour with other drugs due to competition and substitution. Less prescribed drugs may develop independently from external events and have fewer drugs influencing them.

With this knowledge, prescribing patterns can be better understood by assessing which drugs influence each other and which substances are more resilient to external factors. This is important for understanding the consequences of a change in the consumption of one antibacterial drug on another or the impact of a sudden event on antibacterial consumption, such as the COVID pandemic (Romaszko-Wojtowicz et al. 2024). As an extension of future predictions of consumption trends (Bindel and Seifert 2025), insight into prescribing patterns helps to optimise measures for demand planning and to support rational prescribing behaviour in order to maintain the effectiveness of antibacterial therapy (Höhn 2024; Gießelmann 2024).

## Materials and methods

### Data collection

The analysis of consumption and costs per defined daily dose for each antibacterial drugs is based on the ‘Arzneiverordnungsreport’ (AVR, Drug prescription report) for the years 1985 to 2022 (Schwabe and Paffrath 1985, 1986, 1987, 1988, 1991, 1992, 1993, 1994, 1995, 1996, 1998, 1999, 2000, 2001, 2002, 2003, 2004, 2006, 2007, 2008, 2010a, 2010b, 2011a, 2011b, 2012, 2013, 2014, 2015, 2016, 2017; Schwabe et al. 1989, 199,0; Schwabe, 1997; Schwabe et al. 2018, 201,9; Schwabe and Ludwig 2020; Ludwig et al. 2021, 202,3, 2024). The ‘Arzneiverordnungsreport’ is an annual publication of prescription data from the statutory health insurance for 70 million people in Germany for the outpatient sector. Covered ambulant data accounts for around 85% of the total antibacterial consumption of Germany (Bätzing-Feigenbaum et al. 2016). It does not include consumption of inpatient care, private health insurance or private prescriptions (Ludwig et al. 2024; WIdO 2022), introducing a data gap of 15% consumption. Worth noting is the methodological change that took place in the 1990 s. Before 1991, only prescription data from West Germany were collected. Due to the reunification of Germany, prescription data from Eastern Germany have been included since 1991 (Schwabe and Paffrath 1992).

Since we focused on outpatient consumption, only the general chapter “Antibiotika und Chemotherapeutika” (antibiotics and chemotherapeutics) was considered. Therefore, specialised subchapters like urology, dermatology and ophthalmology were excluded.

### Selection of drugs

In order to obtain a general overview, data were collected on chapters that included only antibacterial drugs. This resulted in the exclusion of groups like antimycotics, antiretrovirals and antivirals. Of particular interest were the most commonly prescribed antibacterial drugs. Therefore, the 15 most frequently prescribed drugs (TOP15) were selected for the analysis, based on consumption data from the year 2022.

### Preparation of data

After collecting data for prescriptions defined daily dose, SPSS was used to analyse the correlations between the individual consumption of the 15 drugs. A bivariate correlation analysis was performed using the Pearson correlation coefficient. The coefficient of determination (*R*^2^) was calculated manually. Results were processed and visualised using SPSS and Excel.

### Analysis of the correlations and presentation of data

Correlation analysis focused on three key aspects: statistical significance, direction (positive vs. negative) and strength. The Pearson coefficient (*r*) measures the linear relationship between two variables that are normally distributed. The coefficient ranges between − 1 to + 1. A positive value indicates a direct relationship (both variables increase or decrease similarly), while a negative coefficient indicates an inverse relationship. A coefficient of 0 indicates no linear relationship, while a value of 1 indicates a perfect linear relationship (Mukaka 2012; Newcastle University 2025). We define a correlation above (+/−) 0.8 as strong (Newcastle University 2025), indicating a substantial association between the two variables. Values below 0.8 indicate a weaker relationship.

Besides the correlation coefficient, statistical significance was assessed to determine whether the observed correlations are likely to be generalisable rather than random. The significance level (*p*-value) quantifies the probability of obtaining the observed correlation occurring by coincidence. Only significant values validate the correlation coefficient and allow conclusions to be drawn. If there is no significance, the values are of limited informative power. A value with a significance level of 0.01 and 0.05 is considered significant (Tenny and Abdelgawad 2023).

The coefficient of determination (*R*^2^), calculated by squaring the Pearson correlation coefficient, measures the percentage of variation in the dependent variable that can be attributed to the independent variable, also known as the explanatory power of the correlation. Its value ranges between 0 and 1, with a high value indicating that data points are clustered close to the regression line (Pakay 2023). In addition to significance, it can be used as an indicator of whether the given correlation is valid.

**Fig. 1:**
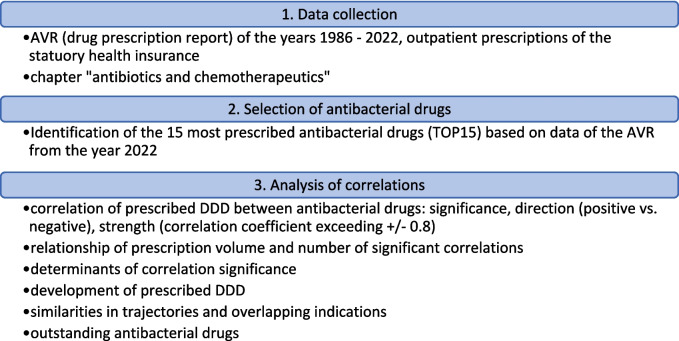
Methodological approach of the selection of antibacterial drugs and analysis of correlations regarding consumption volumes

In Fig. 1, the methodological process is illustrated.

## Results and discussion

### Similarities in antibacterial drug trajectories

The consumption patterns of antibacterial drugs can be characterised by their trajectories over time. Comparisons of consumption trends allow to identify similarities. Three main characteristics of consumption corves are in focus: the decade of peak popularity, the variability of consumption (stable or fluctuating) and the overall trend (increasing, decreasing or fluctuating). The greater the overlap in characteristics, the stronger the similarity between the drugs. Correlation analysis of the consumption is used to confirm and objectify recognised patterns (Table 1 and S1, Figures S1-S15).

**Table 1:**
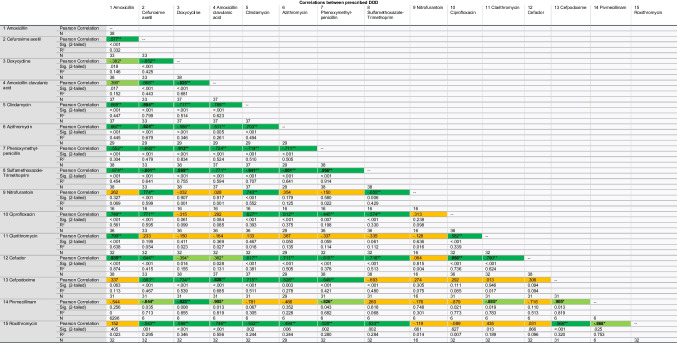
Correlation between prescribed DDD for the 15 most prescribed antibacterial substances (TOP15) in 2022. Dark green colour and “**” highlights a significant correlation for consumption volumes at the 0.01 level, indicating that there is a relation between the consumption trajectories which is not random. Light green colour and “*” highlights a significant correlation at the 0.05 level, indicating that the relation between the consumptions is not random but has a greater uncertainty compared to the 0.01 level. Orange colour indicates a non-significant correlation. Significant strong correlations, defined as a correlation coefficient exceeding (+/−) 0.8, are written in a bold font

**Fig. 2:**
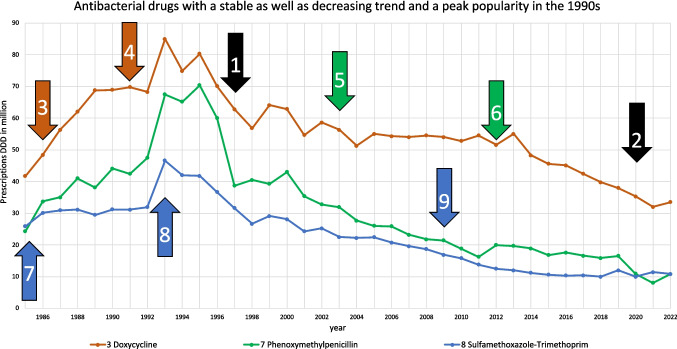
Consumption trajectories and events influencing consumption for the group of antibacterial drugs, which can be described by a stable and decreasing trend, as well as their peak popularity in the 1990 s. Common events are marked with a black arrow, and influences affecting a single drug are highlighted by the colour of its consumption. Common influences include (1) a strong increase in the prescribed DDD of amoxicillin due to a strong decrease in costs (Bindel and Seifert 2024a) and (2) the COVID pandemic. Important influences on doxycycline are (3) decreasing DDD costs and being the cheapest antibacterial drug among the TOP15 (Bindel and Seifert 2024a) and (4) increasing bacterial resistance (Schwabe and Paffrath 1992, 2004). Important influences on phenoxymethylpenicillin are (5) its replacement by amoxicillin in guideline recommendations for community-acquired pneumonia and (6) methodological distortions due to the inclusion of dental prescribed DDD. Important events for sulfamethoxazole-trimethoprim include (7) first-line treatment of urinary tract infections and pneumocystis-carinii pneumonia (Schwabe and Paffrath 1986, 1993), (8) increasing bacterial resistance (Schwabe 1997) and (9) withdrawal of guideline recommendations for empirical treatment of urinary tract infections (Schwabe and Paffrath 2011a)

One prototypical trajectory can be described by a decreasing trend, a stable trajectory and the peak popularity in the 1990 s (Tables 2, 3 and S2-S3). This development is depicted for doxycycline, phenoxymethylpenicillin and sulfamethoxazole-trimethoprim (Fig. 2). As they share these characteristics, their consumption trends are considered to be similar. This is also reflected in their correlation, where all three drugs have a significantly strong positive correlation with each other (Table 1 and S3). In addition to consumption trends, these drugs share characteristics such as overlapping indications in respiratory or urinary tract infections, as more detailed described in Tables 3 and 4. Doxycycline is often prescribed as an alternative for respiratory tract infections in patients with penicillin allergies or infections caused by atypical pathogens (KBV 2024). Phenoxymethylpenicillin remains the first-line treatment for streptococcal pharyngitis, while sulfamethoxazole-trimethoprim has historically been used for gram-negative respiratory infections (Kot 2019) and uncomplicated urinary tract infections (O’Grady 1983). In the past years, all have been increasingly replaced by other substances, such as amoxicillin, due to guideline changes, lower DDD costs or increasing bacterial resistance (Ludwig et al. 2024; DAZ 2012; Karlowsky et al. 2002; Bindel and Seifert 2024a; Schwabe and Paffrath 1992). The decline in doxycycline was due to increasing bacterial resistance and replacement by amoxicillin to tetracyclines in general around 1990, and also to increasing bacterial resistance in *E. coli* around 2004 (Schwabe and Paffrath 1992, 2004; Bindel and Seifert 2024a). Phenoxymethylpenicillin was replaced by amoxicillin due to lower DDD costs and the replacement by amoxicillin in guidelines (Schwabe and Paffrath 2004). Sulfamethoxazole-trimethoprim experienced a substantial decline since the 1990 s due to the popularity of amoxicillin, while increasing resistance in *E. coli* led to its withdrawal for empiric treatment of urinary tract infections in the late 2000 s (Karlowsky et al. 2002; Schwabe and Paffrath 2002, 2003, 2011a, b). These changes contributed to their similarly declining trends.

**Table 2:**
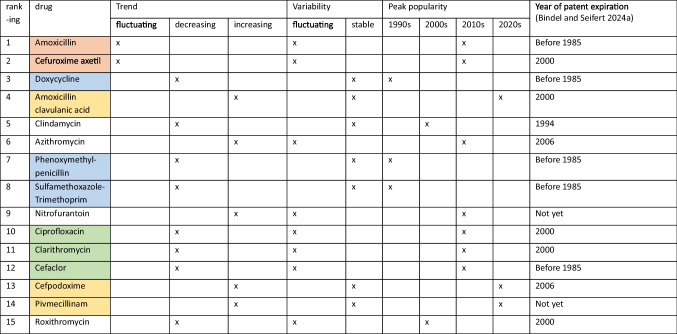
Key characteristics for consumption trajectories. Considered characteristics include peak popularity (years with the largest consumption volume), variability of the consumption (strong versus slight changes between single years), trend (comparison between the first and latest consumption volume) and year of patent expiration. Antibacterial drugs that share a similar trajectory are highlighted in the same colours

**Table 3:** Grouping of consumption trajectories for the analysed antibacterial drugs which show strong similarity. The similar characteristics of their consumption as well as overlapping indications are mentioned

Drugs with large similarities	Trend	Variability	Peak popularity	Overlapping indication (Ewig et al. 2021; KBV 2024; EAU 2024; Bindel and Seifert 2024a)
Amoxicillin-clavulanic acid, cefpodoxime, pivmecillinam	Increasing	Stable	2020 s	Respiratory tract infections, urinary tract infections
Doxycycline, phenoxymethylpenicillin, sulfamethoxazole-trimethoprim	Decreasing	Stable	1990 s	Respiratory tract infections
Ciprofloxacin, clarithromycin, cefaclor	Decreasing	Fluctuating	2010 s	Respiratory tract infections
Amoxicillin, cefuroxime axetil	Fluctuating	Fluctuating	2010 s	Respiratory tract infections, especially when resistance or allergies limit options

**Table 4:** Indication spectrum of the fifteen most used antibacterial substances. Mentioned are the indications, suitable pathogens and bacterial resistance trends. Drugs with shared indications are listed, where an explanation is given in brackets. Furthermore, usage notes for the antibacterial drugs are given, referring to the trajectory of consumption, bacterial resistance issues and usage fields. The used source for each category is given in parentheses

Ranking	Antibacterial substance	Primary indications (Bindel and Seifert 2024a, 2024d; Ewig et al. 2016, 2020, 2021, 2024; EAU 2024; AWMF 2018, 2019a, 2019b, 2020, 2023, 2024)	Key pathogens (Bindel and Seifert 2024a, 2024d)	Resistance trends (Bindel and Seifert 2024d; RKI 2024)	Drugs with shared indications (Bindel and Seifert 2024d)	Usage notes (Bindel and Seifert 2024a, 2024d)
1	Amoxicillin	Respiratory, ENT and skin infections	*S. pneumoniae, H. influenzae, E. coli, E. faecalis*	Rising	Amoxicillin-clavulanic acid (similar indications, alternative for resistant pathogens), cefuroxime axetil (effective for similar pathogens), cefaclor (class-based similarity, cephalosporin alternative), azithromycin (alternative for penicillin-allergic patients)	Frequently prescribed first-line treatment, cost-sensitive
2	Cefuroxime axetil	Respiratory and urinary infections	*E. coli, K. pneumoniae, S. pneumoniae*	Rising	Amoxicillin (similar indications, alternative treatment for respiratory infections), clindamycin (effective for resistant *S. pneumoniae* infections), cefaclor (class-based similarity, cephalosporin alternative), azithromycin (alternative for respiratory infections), cefpodoxime (class-based similarity, cephalosporin alternative)	Broad-spectrum, resistance increasing due to higher usage
3	Doxycycline	Acne, respiratory and Lyme disease	*S. pneumoniae, S. aureus*	Declining	Azithromycin (alternative treatment for respiratory infections), roxithromycin (class-based similarity, both are macrolides), clarithromycin (alternative for respiratory infections)	Declining resistance due to decreased use
4	Amoxicillin-clavulanic acid	Complicated respiratory and urinary infections	*S. aureus, P. mirabilis, K. pneumoniae*	Rising	Amoxicillin (similar indications, enhanced activity via clavulanic acid), cefuroxime axetil (effective for similar pathogens), clindamycin (alternative treatment)	Effective against resistant strains of Amoxicillin; widely used despite rising resistance
5	Clindamycin	Bone, joint and dental infections	*S. aureus, anaerobic bacteria*	Declining	Cefuroxime axetil (effective for resistant *S. pneumoniae* infections), amoxicillin-clavulanic acid (effective for similar pathogens), azithromycin (alternative treatment for skin infections)	Alternative for penicillin-allergic patients, only targeted use in specific conditions indicated
6	Azithromycin	Respiratory infections	*S. pneumoniae*	Declining	Amoxicillin (treatment for respiratory infections), doxycycline (alternative treatment for respiratory infections), amoxicillin clavulanic acid (alternative treatment for respiratory infections), clarithromycin (class-based similarity, both macrolides), roxithromycin (class-based similarity, macrolide alternative) (Bindel and Seifert 2024d)	Vaccination efforts reduced infections and bacterial resistance; STIKO guideline recommendation impacted use
7	Phenoxymethyl-penicillin	Respiratory, ENT and dental infections	*S. pneumoniae, S. pyogenes*	Rising	Amoxicillin (similar indications for respiratory infections, broader spectrum), clindamycin (alternative for penicillin-allergic patients), cefaclor (similar indications for respiratory infections)	Commonly used for mild respiratory infections and dental infections, often substituted with Amoxicillin in severe infections
8	Sulfamethoxazole-trimethoprim	Urinary tract, gastrointestinal and respiratory infections	*E. coli, K. pneumoniae, P. mirabilis*	Rising	Nitrofurantoin, pivmecillinam (similar indications, urinary infections)	Resistance growing, especially for *E. coli*; still widely used for UTIs
9	Nitrofurantoin	Urinary tract infections	*E. coli, E. faecalis*	Rising	Sulfamethoxazole-trimethoprim, pivmecillinam (similar indications, urinary tract infections)	Resistance concerns increasing with rising consumption
10	Ciprofloxacin	Complicated urinary and gastrointestinal infections	*K. pneumoniae, P. aeruginosa*	Mixed	Sulfamethoxazole-trimethoprim (effective for similar pathogens), nitrofurantoin (effective for urinary tract infections)	Resistance varies across pathogens, only indicated for severe infections
11	Clarithromycin	Respiratory and gastrointestinal infections	*S. pneumoniae, H. pylori*	Declining	Azithromycin (similar indications, macrolide alternative), roxithromycin (class-based similarity, macrolide alternative)	Effective for *H. pylori* treatment; declining resistance due to decreased use in the last years
12	Cefaclor	Respiratory and urinary infections	*E. coli, S. pneumoniae*	Declining	Amoxicillin (similar indications, respiratory infections), cefuroxime axetil (class-based similarity, cephalosporin alternative)	Used less frequently due to newer generations of cephalosporins
13	Cefpodoxime	Respiratory and skin infections	*E. coli, K. pneumoniae*	Rising	Cefuroxime axetil, cefaclor (class-based similarity, cephalosporin alternative)	Preferred for penicillin-allergic patients, rising use linked to resistance
14	Pivmecillinam	Urinary tract infections	*E. coli, K. pneumoniae*	Rising	Nitrofurantoin (effective for urinary tract infections), sulfamethoxazole-trimethoprim (effective for similar pathogens)	Used as a first-line treatment in Europe for uncomplicated cystitis, but rising resistance in *E. coli*
15	Roxithromycin	Respiratory and skin infections	*S. pneumoniae*	Declining	Clarithromycin (class-based similarity, macrolide class-based similarity), azithromycin (effective for similar pathogens, macrolide alternative)	Used less frequently due to newer generations of macrolides

**Fig. 3:**
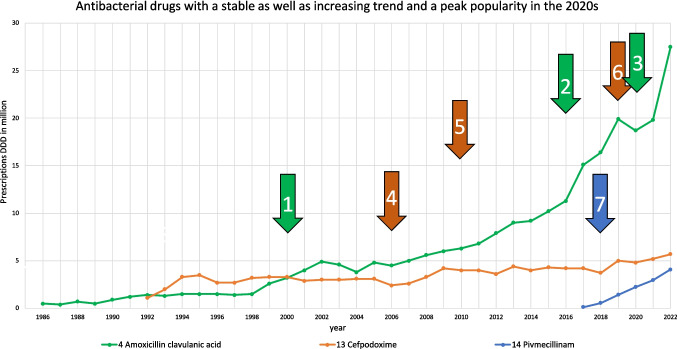
Consumption trajectories and events influencing consumption for the group of antibacterial drugs, which can be described by a stable and increasing trend, as well as their peak popularity in the 2020 s. Events affecting a single drug are highlighted by the colour of its consumption. Important influences on amoxicillin clavulanic acid include (1) patent expiration and declining DDD costs, (2) first choice for complicated community-acquired pneumonia (Ewig et al. 2016) and (3) indication for recurrent sinusitis and otitis media (Ewig et al. 2021; Ludwig et al. 2021). Important events for cefpodoxime include (4) patent expiration and declining DDD costs, (5) indication for alternative in treatment of UTIs (S3 guideline 2010) and (6) indication for uncomplicated cystitis and gonorrhoea (Schwabe et al. 2019). An important influence on pivmecillinam is (7) indication for uncomplicated cystitis (Schwabe et al. 2019)

An increasing trend, a stable trajectory and a peak popularity in the 2020 s is another recognised trajectory (Fig. 3). Amoxicillin clavulanic acid, cefpodoxime and pivmecillinam form this group. Their shared similarities also result in significantly strong positive correlations with each other. Reasons for the common increasing trend can be found in the treatment recommendations of recent guidelines, e.g. for urinary tract infections (Wagenlehner et al. 2024) (Tables 3 and 4). Amoxicillin clavulanic acid and cefpodoxime are also widely used in respiratory tract infections due to their effectiveness against beta-lactamase-producing pathogens (PEG 2019). Pivmecillinam, although more specific to urinary tract infections, has gained popularity for its efficacy against resistant gram-negative bacteria like *E. coli* (Fuchs and Hamprecht 2019; Kresken et al. 2022). Amoxicillin-clavulanic acid began to increase in use after its patent expired and its price fell sharply around 2000 (Bindel and Seifert 2024a), accelerating after it became the first choice for complicated community-acquired pneumonia in 2016 (Ewig et al. 2016) and for recurrent sinusitis and otitis media since 2020 (Ewig et al. 2021; Ludwig et al. 2021). Cefpodoxime went off-patent in 2006, followed by a sharp price decline and an increase in consumption (Bindel and Seifert 2024a). In 2010, it was named as an alternative in the treatment of urinary tract infections (S3 guideline 2010) and has recently been increasingly used in the treatment of syphilis and gonorrhoea (Schwabe and Ludwig 2020). Pivmecillinam was launched around 2017, indicated for uncomplicated cystitis in 2018 (Schwabe et al. 2019; Kaye et al. 2025), and shown to be effective against ESBL-producing gram-negative bacteria (Fuchs and Hamprecht 2019). These factors contributed to their similar increasing trends.

**Fig. 4:**
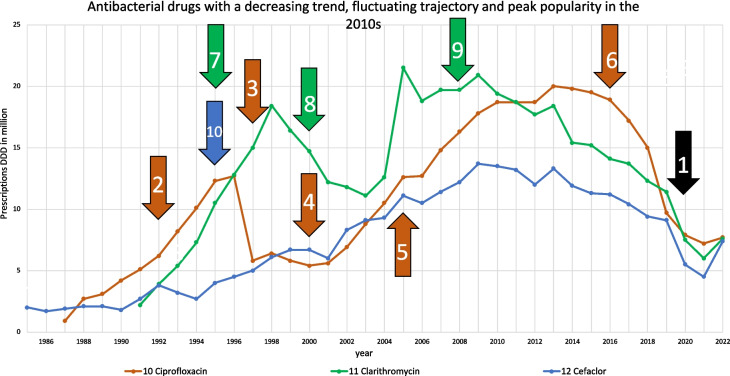
Consumption trajectories and events influencing consumption for the group of antibacterial drugs, which can be described by a decreasing trend, fluctuating consumption trajectory as well as their peak popularity in the 2010 s. Common events are marked with a black arrow, and influences affecting a single drug are highlighted by the colour of its consumption. Common events include (1) the COVID pandemic. Important influences on ciprofloxacin include (2) concerns about side effects of fluoroquinolones (Schwabe and Paffrath 1992), (3) strong increase in the consumption of amoxicillin, (4) patent expiration and price drop, (5) withdrawal from the indication of community-acquired pneumonia (Schwabe and Paffrath 2008) and (6) restrictions in use due to severe side effects like tendonitis and QT prolongation (RKI 2023). Important events for clarithromycin are (7) being a component in triple therapy of H. pylori (Schwabe and Paffrath 1996), (8) patent expiration and price drop and (9) increasing bacterial resistance (Schwabe and Paffrath 2006; Bluemel et al. 2020). For cefaclor, an important event is considered to be (10) decreasing costs

As having a decreasing trend, fluctuating trajectory and peak popularity in the 2010 s, ciprofloxacin, clarithromycin and cefaclor can be described (Fig. 4 and Table 2). Their similarities are confirmed by the correlation of their consumption (Table 1), with all having significantly positive correlations, while ciprofloxacin and cefaclor are even strongly correlated. All these antibacterial drugs have declined in their popularity due to increases in bacterial resistance and their replacement by other agents in treatment (Tables 3 and 4). Ciprofloxacin has seen an increase in consumption since 2000 because of its patent expiration and a price drop (Bindel and Seifert 2024a). Severe side effects such as tendonitis and QT prolongation of fluoroquinolones (Shu et al. 2022; Falagas et al. 2007) led to a restriction of use from 2019 (RKI 2023), followed by a sharp decline in ciprofloxacin consumption. Rising bacterial resistance has contributed to its decline (Schwabe and Paffrath 2008; PEG 2016). Clarithromycin, despite its importance in *H. pylori* eradication regimens since the 1990 s (Schwabe and Paffrath 1996), has faced declining consumption due to increasing bacterial resistance, particularly for *Haemophilus* around 2006 (Schwabe and Paffrath 2006) and *H. pylori* around 2018 (Bluemel et al. 2020). Cefaclor, a second-generation cephalosporin, has largely been replaced by newer cephalosporins with broader spectra and improved resistance profiles (Bindel and Seifert 2024a).

**Fig. 5:**
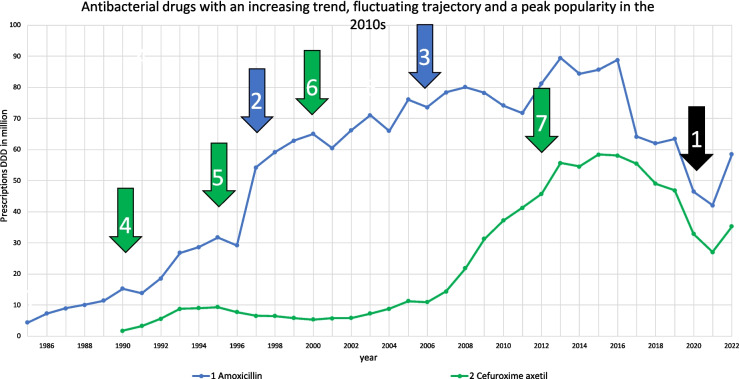
Consumption trajectories and events influencing consumption for the group of antibacterial drugs, which can be described by an increasing trend, fluctuating consumption trajectory and their peak popularity in the 2010 s. Common events are marked with a black arrow, and influences affecting a single drug are highlighted by the colour of its consumption. Common events include (1) the COVID pandemic. Important influences on amoxicillin are (2) strong price drop and (3) first choice in guidelines for community-acquired pneumonia (Ewig et al. 2016). Important events for cefuroxime axetil are (4) replacement of cefaclor due to efficacy in treatment (Bindel and Seifert 2024a), (5) increase of cefaclor due to cheaper DDD costs, (6) patent expiration and strong price drop and (7) withdrawal as treatment recommendation for community-acquired pneumonia (Bätzing-Feigenbaum et al. 2016)

Another common trajectory can be described by an increasing trend, fluctuating trajectories and a peak of popularity in the 2010 s (Table 2). Amoxicillin and cefuroxime axetil form this smaller but notable pair (Fig. 5), with the similar trend also reflected in a significantly positive correlation and similar characteristics (Tables 2, 3 and S3). Both are commonly prescribed for respiratory tract infections (Ewig et al. 2021). Amoxicillin rose sharply after its patent expired and its DDD costs fell around 1997 (Bindel and Seifert 2024a). By 2005, it had become the first-line treatment for community-acquired pneumonia (Ewig et al. 2016). Similarly to amoxicillin, cefuroxime axetil experienced a sharp increase after patent expiry and large price reductions in the 2000 s (Bindel and Seifert 2024a). In recent years, both drugs have experienced a decline due to COVID effects (Romaszko-Wojtowicz et al. 2024), in the case of amoxicillin due to replacement by amoxicillin-clavulanic acid (Bindel and Seifert 2024a), and in the case of cefuroxime axetil due to its withdrawal from guideline recommendations as first-line treatment for community-acquired pneumonia (Bätzing-Feigenbaum et al. 2016).

There are trends that are present in most of the drugs. Around the 1990 s, most antibacterial substances experienced an increase in consumption. The reasons are now fully understood, but overall declining DDD costs (Bindel and Seifert 2024a) as well as the German reunification with a methodological change by including consumption data for eastern Germany (Schwabe and Paffrath 1992) may have played a role and contributed to the increase. From 2010 onwards, a decline in outpatient consumption data is observed (Schwabe et al. 2019; Scholle et al. 2022; Holstiege et al. 2020a), a trend that still persists in the sum of consumptions (Bindel and Seifert 2024a). In 2020 and 2021, there is a sudden decrease for most substances due to the COVID pandemic (Selke Krulichová et al. 2022). This is followed by an increase in 2022, approaching pre-pandemic levels (Koh et al. 2025; Ludwig et al. 2024).

Summarised, antibacterial drugs can be classified into distinct groups based on their historical development in consumption (Figs. 2, 3, 4, 5 and Table 2). The similarities are reflected and confirmed by strong correlations between these drugs, further reinforcing the robustness of these patterns. These patterns are often consistent with their use in specific infections and their role in treatment guidelines, highlighting the interplay between overlapping indications, competition and prescribing behaviour over time (Tables 3 and 4).

### Determinants of consumption patterns and relation to correlations between antibacterial drugs

**Fig. 6:**
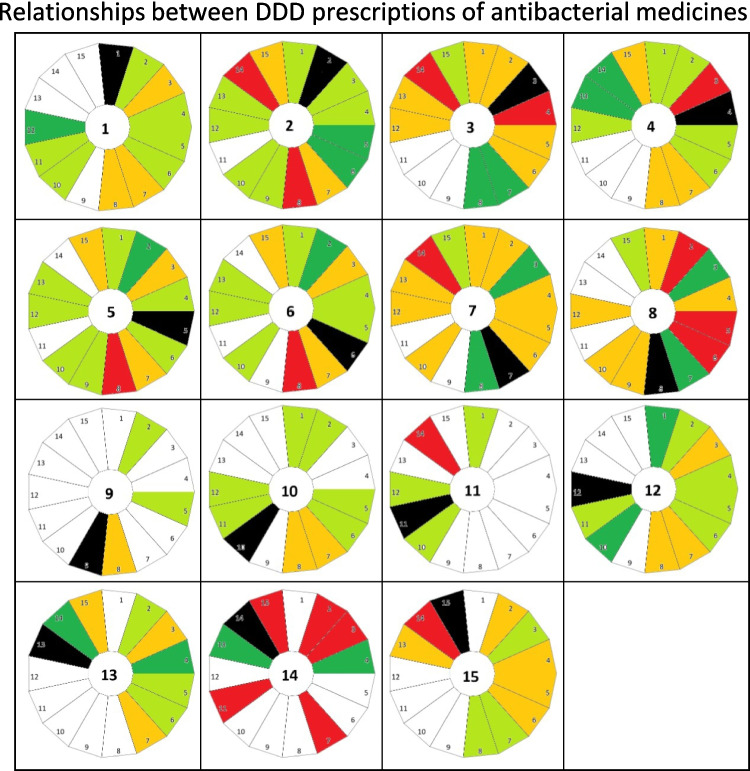
Overview of the relationships between the consumption of antibacterial drugs. Each antibacterial drug is represented by a circle. The ranking number of the reference antibacterial is in the centre, while the compared substance is represented by its number in an outer sector. Black colour marks the sector of the reference itself. Light green highlights significant positive correlations; dark green highlights significant strong positive correlations. Orange highlights significant negative correlations; red highlights significant strong negative correlations. White highlights a non-significant correlation. Antibacterial drugs are ranked by the following order: amoxicillin (1), cefuroxime axetil (2), doxycycline (3), amoxicillin clavulanic acid (4), clindamycin (5), azithromycin (6), phenoxymethylpenicillin (7), sulfamethoxazole-trimethoprim (8), nitrofurantoin (9), ciprofloxacin (10), clarithroymcin (11), cefaclor (12), cefpodoxime (13), pivmecillinam (14) and roxithromycin (15)

**Fig. 7:**
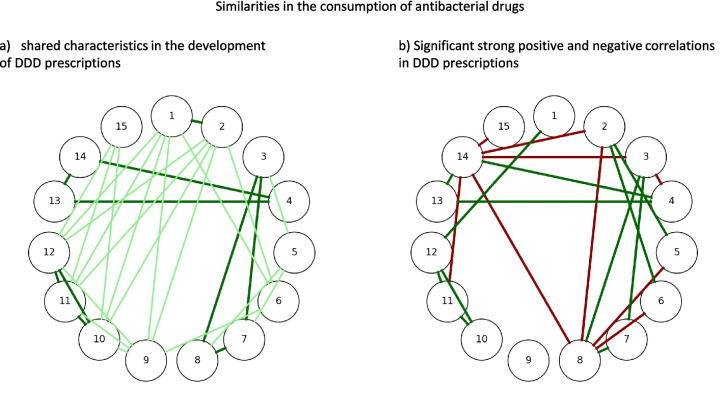
Similarities in the consumption patterns of antibacterial drugs. Depicted is in **a** the shared characteristics in the development of prescribed DDD, based on Table 3, S1 and S2. In dark green, three shared characteristics are highlighted, while in light green, two shared patterns are marked. In **b**, significant strong positive and negative correlations are displayed, positive correlations in green colour and negative correlations in red colour. The following antibacterial drugs are depicted: amoxicillin (1), cefuroxime axetil (2), doxycycline (3), amoxicillin clavulanic acid (4), clindamycin (5), azithromycin (6), phenoxymethylpenicillin (7), sulfamethoxazole-trimethoprim (8), nitrofurantoin (9), ciprofloxacin (10), clarithroymcin (11), cefaclor (12), cefpodoxime (13), pivmecillinam (14) and roxithromycin (15). Notably, the groups of three with similar characteristics display also strong correlations towards each other

**Fig. 8:**
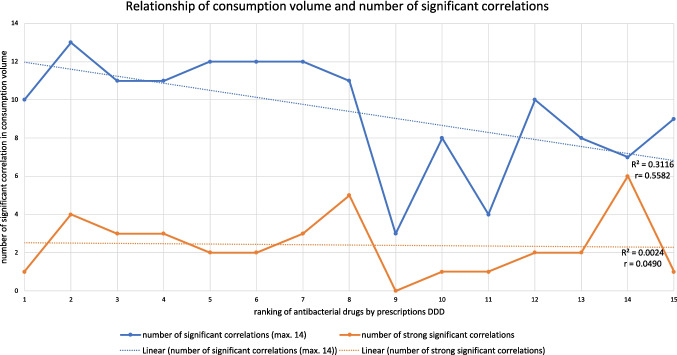
Relationship of consumption volume and number of significant correlations for antibacterial substances. Considered is the number of significant and the number of strong significant correlations within prescribed DDD. For each curve, a trend line is included to illustrate the trend depending on the ranking. The following antibacterial drugs are depicted: amoxicillin (1), cefuroxime axetil (2), doxycycline (3), amoxicillin clavulanic acid (4), clindamycin (5), azithromycin (6), phenoxymethylpenicillin (7), sulfamethoxazole-trimethoprim (8), nitrofurantoin (9), ciprofloxacin (10), clarithroymcin (11), cefaclor (12), cefpodoxime (13), pivmecillinam (14) and roxithromycin (15)

The significance of correlations between antibacterial substances provides valuable insights into the dynamics of their consumption patterns, revealing independent behaviours and potential interdependencies (Tables 5 and 6). Explanations for the appearance of significantly strong correlations are often overlapping indications and substitution by/of another drug over time (Table 5). Shared patterns in the development of consumption are often reflected in significant correlations, either positive—where two substances follow similar—or negative, indicating opposing trends (Figs. 6 and 7). For instance, amoxicillin and phenoxymethylpenicillin show a strong negative correlation: as the consumption of amoxicillin has risen, phenoxymethylpenicillin has steadily declined (Fig. 8). There is evidence that these correlations are not coincidental but that the two drugs influence each other, for example the replacement of phenoxymethylpenicillin by amoxicillin in many indications (Bindel and Seifert 2024a; Skarpeid and Høye 2018).

**Table 5:** Overview of significantly strong correlations, offering a possible explanation why the consumption trajectories are related to each other. For each strongly correlated drug, the direction of correlation is given in brackets, which is either positive (similar trends) or negative (opposing trends)

Ranking	Antibacterial substance	Strongly correlated antibacterial substances	Possible explanation
1	Amoxicillin	Cefaclor (positive)	Shared use in respiratory infections and similar consumption trends during peak usage periods
2	Cefuroxime axetil	Clindamycin (positive), azithromycin (positive)	Overlapping indications for respiratory and skin infections during the 2000 s
3	Doxycycline	Phenoxymethylpenicillin (positive), sulfamethoxazole-trimethoprim (positive)	Shared usage in treating respiratory infections and declining trend
4	Amoxicillin-clavulanic acid	Cefpodoxime (positive), pivmecillinam (positive)	Shared indication of complicated respiratory and urinary infection, increased use due to decreasing DDD costs
5	Clindamycin	Cefuroxime axetil (positive), sulfamethoxazole-trimethoprim (negative)	Strong positive correlation with cefuroxime axetil, driven by shared usage in skin and soft tissue infections, similar trends in development of prescribed DDD; negative correlation with sulfamethoxazole-trimethoprim likely due to diverging trends in consumption
6	Azithromycin	Cefuroxime axetil (positive), sulfamethoxazole-trimethoprim (negative)	Strong positive correlation with cefuroxime axetil, reflecting shared trends in treating respiratory infections from 2007 to 2013; negative correlation with sulfamethoxazole-trimethoprim likely due to diverging trends in consumption (increasing vs. decreasing)
7	Phenoxymethyl-penicillin	Doxycycline (positive), sulfamethoxazole-trimethoprim (positive), pivmecillinam (negative)	Shared focus on respiratory infections and overlapping historical prescribing trends with positively correlated drugs; opposing trends in consumption patterns with pivmecillinam (decreasing vs. increasing)
8	Sulfamethoxazole-trimethoprim	Cefuroxime axetil (positive), doxycycline (positive), clindamycin (negative), azithromycin (negative), phenoxymethylpenicillin (positive), pivmecillinam (negative)	Reflecting shared indications and replacement by amoxicillin for respiratory infections, shared trends in prescribed DDD; opposing trends in consumption patterns with pivmecillinam (decreasing vs. increasing)
9	Nitrofurantoin	None (no significantly strong correlations)	Likely due to distinct role in treating uncomplicated urinary tract infections; niche indication
10	Ciprofloxacin	Cefaclor (positive)	Overlapping use in treating respiratory and urinary infections during certain periods, similar development of prescribed DDD
11	Clarithromycin	Pivmecillinam (negative)	opposing trend in prescribed DDD due to non-shared indications (declining vs. increasing)
12	Cefaclor	Amoxicillin (positive), ciprofloxacin (positive)	Shared indications for respiratory infections and aligned consumption trends
13	Cefpodoxime	Amoxicillin-clavulanic acid (positive)	Shared use in respiratory and urinary infections, shared increasing trend
14	Pivmecillinam	Amoxicillin-clavulanic acid (positive), cefpodoxime (positive), pivmecillinam (positive)	Shared trends in treating urinary and respiratory infections, overlapping periods of increased use
15	Roxithromycin	Pivmecillinam (negative)	Opposing trend in prescribed DDD due to non-shared indications (declining vs. increasing)

**Table 6:** Discussion of medical causes for the characteristic of antibacterial drugs that seem less influenced by consumption trends, which is indicated by the comparably low number of significant correlations

Ranking	Antibacterial substance	Use field (Bindel and Seifert 2024a)	Reason for independence	Explanation (Bindel and Seifert 2024a, 2024d)
9	Nitrofurantoin	Uncomplicated urinary tract infections	Restricted application spectrum; niche indication	Narrow-spectrum activity, effective against lower urinary tract pathogens; rarely prescribed for other infections; minimal overlap with antibacterial drugs targeting respiratory or systemic infections
14	Pivmecillinam			
11	Clarithromycin	Respiratory infections, particularly atypical pneumonia and *Helicobacter pylori* eradication	Specific use in atypical respiratory infections and *H. pylori* eradication therapy; not commonly used as a broad-spectrum macrolide	Increasing resistance among macrolide-targeted pathogens (e.g. *S. pneumoniae*) limits widespread use; usage has shifted to highly specific indications such as triple therapy for* H. pylori*

Common historical events can also affect antibacterial consumption for many drugs. Some events, such as guideline changes, affects predominantly related drugs and can lead to substitution of one drug for another (Tables 4, 5, Figs. 2, 3, 4 and 5), as happened with amoxicillin and phenoxymethylpenicillin, resulting in a significant negative correlation. However, when events or trends occur in most drugs, such as the COVID pandemic (Figs. 8 and 9) or an overall increase (around the 1990 s) or decrease (around the 2010 s) in consumption (Schwabe and Paffrath 1992, 2010b; Ludwig et al. 2024; Selke Krulichová et al. 2022), this can create the appearance of an alignment even among otherwise unrelated substances. Such a coincident uniform trend is present for doxycycline and clarithromycin, which have a non-significant correlation with each other, but whose similar declines during this period were due to the external effects of the pandemic rather than intrinsic relationships.

Interestingly, substances belonging to the same substance class do not consistently exhibit stronger relations, expressed by correlations of their consumption, than those from different classes. While some correlations, such as that between amoxicillin and amoxicillin-clavulanic acid (both aminopenicillins), are significant, others within the same class, like cephalosporins or macrolides, may not be. Among macrolides, correlations can vary from positive to negative, suggesting that shared substance class alone does not guarantee a consistent relationship. This variability may stem from overlapping or distinct indications within a class, as well as the possibility of shared indications across different classes (Tables 3 and 4). For example, clarithromycin decreased because of rising bacterial resistance (Schwabe and Paffrath 2006; Bluemel et al. 2020), while azithromycin increased due to an extended spectrum and lower costs than comparable treatment options (Bindel and Seifert 2024a; Sandman and Iqbal 2024) (Tables 4 and 5).

The presence of significant correlations can be attributed to various factors (Tables 4 and 5) (Bindel and Seifert 2024a; Schröder and Seifert 2024). Competition between substances with overlapping indications may lead to correlations influenced by factors such as DDD costs (Bindel and Seifert 2024b) or side effect profiles. External drivers, such as growing antibacterial resistance (UN 2024; IHME 2024; Salam et al. 2023), changes in clinical guidelines (Ouldali et al. 2017; Thornhill et al. 2011) or restrictions due to safety concerns (EMA 2023), also play a role. Additionally, broader influences such as vaccination campaigns (Buckley et al. 2019; Klugman and Black 2018) or pandemic-related safety measures (Selke Krulichová et al. 2022) can create similar trends. A unique case arises with substances like amoxicillin and amoxicillin-clavulanic acid, where one substance is used both alone and in combination therapy, adding complexity to their relationship.

Prescribing behaviour also has an impact (Rodrigues et al. 2013; Kasse et al. 2024; Merlo et al. 2025; van der Zande et al. 2019). There is evidence of regional differences within the federal states of Germany, as well as a persistent prescribing shift between East and West Germany (Bätzing-Feigenbaum et al. 2016; Augustin et al. 2015; Holstiege et al. 2020a; Scholle et al. 2022). Around 2018, the prevalence of antibacterial treatment was much higher in West Germany than in East Germany, being observed in all age groups (Holstiege et al. 2020a; Scholle et al. 2022). While an overall decreasing trend is reported, eastern Germany showed a strong decrease in use compared to the west between 2010 and 2018 (Augustin et al. 2015; Holstiege et al. 2020a, 2020b). These differences can only be attributed to socio-cultural developments and cultural behaviour, as the prevalence of infection doesn’t vary (Augustin et al. 2015; Scholle et al. 2022). Unfortunately, the consumption data used do not differentiate by region, age or specialisation, so these determinants cannot be observed in the analyses.

Conversely, non-significant correlations are more likely when substances are prescribed for unrelated conditions or when their time trends do not to overlap. Temporal disparities in their popularity, as well as external influences affecting only one substance, such as adverse study results or supply shortages can further contribute to their non-significance.

### Outstanding antibacterial substances

The analysis of correlations among prescribed DDD reveals notable characteristics for certain antibacterial substances. This section explores these substances, focusing on consumption patterns and determinants for the largest and smallest numbers of significant correlations, the largest number of strong correlations and the largest proportion of positive significant correlations (Tables 4, 5 and 6).

The largest number of significant correlations is provided by cefuroxime axetil (13), followed closely by clindamycin (12), azithromycin (12) and phenoxymethylpenicillin (12) (Table S1). Both cefuroxime axetil and clindamycin also show the highest number of significant positive correlations, each with five. These patterns can be attributed to common trends in antibacterial drug use, characterised by periods of increasing popularity followed by decline. Overall trends, including a general increase until 2010, a subsequent decline and a sudden drop due to the COVID pandemic, are seen for many antibacterial drugs and are also apparent when looking at total antibacterial drug consumption (Selke Krulichová et al. 2022). A typical trajectory for a single drug can be described by a sudden increase and peak of popularity followed by a steady decline (Bindel and Seifert 2024a). Cefuroxime axetil was very popular around the 2010 s, while phenoxymethylpenicillin peaked in the 1990 s. For cefuroxime axetil, its trajectory being prototypical and aligning with general trends in consumption may explain the large number of correlations. Phenoxymethylpenicillin decreased simultaneously while other drugs increased and replaced phenoxymethylpenicillin, as reflected in a large number of significant negative correlations. Azithromycin, on the other hand, showed a steady increase in consumption and may have maintained a consistent relationship with other declining antibacterials. For clindamycin, the correlations should be interpreted with caution, as a significant distortion occurred in 2012. Nevertheless, its overall trajectory is consistent with a general decline.

The largest number of strong correlations are exhibited by pivmecillinam (6) and sulfamethoxazole-trimethoprim (5) (Figs. 7 and 10). Interestingly, pivmecillinam, despite its strong correlations, exhibits relatively few significant correlations overall (7). As a relatively recently introduced drug, pivmecillinam has shown a steady upward trend, correlating significantly with drugs that follow similarly stable developments—whether increasing or decreasing. Furthermore, pivmecillinam is only indicated for urinary tract infections (Wagenlehner et al. 2008; Dewar et al. 2013), which limits overlapping indications with other substances. Sulfamethoxazole-trimethoprim, on the other hand, has been in steady decline since its peak popularity in the late 1990 s, showing strong correlations with substances that also exhibit particularly consistent patterns of increase or decrease over time.

In contrast, the lowest number of significant correlations is exhibited by nitrofurantoin (3) and clarithromycin (4) (Table 6). Nitrofurantoin, another relatively new drug, has experienced an upward trend since its introduction. Its short observation period, combined with inconsistent growth patterns, likely accounts for the limited number of significant correlations. Clarithromycin exhibits an unsteady increase with extended periods of slow increase until 2005, followed by a decline. This inconsistent growth, contrasted with the sharper rises and falls of other substances, may explain its limited correlations. Additionally, clarithromycin’s peak popularity occurred later than that of many other drugs, during a period when other substances were already declining.

Antibacterial substances with more than eight significant correlations are classified as ‘dependent’ (Table S2 and S3), as their development is associated with many other substances and aligns with overall trends, suggesting a linked relation. In contrast, drugs with fewer than eight significant correlations are considered to be more ‘independent,’ as their development is more atypical compared to other substances, suggesting less influenced use. Nitrofurantoin, clarithromycin and pivmecillinam are considered as independent since their consumption trajectories is different than that from other analysed drugs, reflected in an outstanding low number of significant correlations. These three drugs appear to be used in niche indications such as urinary tract infections or highly specific indications such as the eradication of *H. pylori* (Bindel and Seifert 2024a). In Table 6, an overview about potential medical causes for these substances can be found.

In summary, the more common the pattern of use of a particular antibacterial drug compared to others, the greater the number of significant correlations it tends to show. Strong correlations are particularly evident for drugs with overlapping indications and for stable increases or decreases. A typical pattern for many antibacterial drugs is a sharp rise followed by a long decline, with peaks in popularity in alternating years and to varying degrees. Examples are doxycycline and phenoxymethylpenicillin. In contrast, drugs that deviate from the general trend and develop more independently due to niche indications, have a lower number of significant correlations.

### Hypothesis of a large consumption volume leading to a higher number of significant correlations

It appears that frequently prescribed antibacterial drugs have more significant correlations than others having a lower consumption (Fig. 8 and Table S3). The hypothesis suggesting a link between consumption volume and the number of significant correlations, represented by the ranking of antibacterial drugs, was introduced earlier. This relationship highlights how frequently prescribed drugs tend to exhibit higher and more stable numbers of significant correlations, while less frequently prescribed drugs display greater variability. In the following, this hypothesis is examined and discussed.

At first glance, based on the correlation charts and summary tables, the connection between consumption volume and significant correlations is not immediately apparent (Table S3). However, a closer inspection reveals that the number of significant correlations tends to be higher and more consistent among the top-ranked antibacterial drugs compared to those less often prescribed. The broader trend suggests a relatively stable level of significant correlations for the most frequently prescribed drugs. In contrast, the less frequently prescribed drugs show considerable fluctuations in their number of significant correlations, but at a lower level.

To provide a clearer understanding and visual representation, a chart was created to plot the number of correlations against the ranking of antibacterial drugs (Fig. 8). A trend line was added to highlight the trends. The chart reveals that the number of significant correlations remains relatively steady among the highest-ranked drugs. However, around the midpoint of the rankings, the number of significant correlations begins to decline sharply. Beyond this point, among the less frequently prescribed drugs, the number of significant correlations fluctuates strongly, with some drugs showing very few correlations and others exhibiting high numbers.

This trend is also observable, though less pronounced, in the number of strong correlations, suggesting that consumption volume may serve as an indicator of a drug’s impact on the consumption of others, potentially reflecting therapeutic substitutions. The greater the superiority of a drug over alternatives, the more consumption trajectories it may influence, leading to a higher number of significant correlations. However, the weaker connection between consumption volume and strong correlations indicates that other factors, such as costs, efficacy or guideline recommendations, exert a stronger long-term influence on drug use.

High-ranking drugs are often well-established substances with broad applications, including first-line treatments such as amoxicillin (Bindel and Seifert 2024a, 2024d; Ewig et al. 2016; Ludwig et al. 2024). Their consistently high number of significant correlations likely stems from their established role in recommended treatments and widespread use across clinical settings. In contrast, lower-ranking drugs tend to include newer substances and those indicated for specific pathogens or niche applications. Their greater heterogeneity contributes to the observed fluctuations in correlation patterns. Moreover, drug consumption is shaped by numerous factors beyond interdependencies with other drugs, including the evolution of bacterial resistance (Bindel and Seifert 2024a), the incidence of infectious diseases (Deutsches Ärzteblatt 2025), sudden events such as the COVID pandemic (Selke Krulichová et al. 2022), supply shortages (Lambert et al. 2025) and shifts in prescribing behaviour (Holstiege et al. 2020b). These external influences add complexity to the evaluation, as their extent remains uncertain, making interpretation more challenging. It is unclear whether the relationship between consumption volume and the number of significant correlations reflects a direct association or is instead driven by shared trends and external influences.

## Limitations

The analysis in this study is based on data from the Arzneiverordnungsreport. Data collection for prescribed DDD depended on the structure of the chapters under consideration. Changes in the structure of these sections over the years have led to distortions in the consumption data for clindamycin and phenoxymethylpenicillin in 2012 (Schwabe and Paffrath 2013), as well as a general methodological change due to German reunification and the inclusion of prescribed DDD from East Germany since 1991 (Schwabe and Paffrath 1992). There is a lack of information on patients and physicians, meaning that the data are not further disaggregated by region, age or physician specialisation. Furthermore, the data only include data on outpatient prescribed DDD dispensed by pharmacies of the statutory health insurance (Ludwig et al. 2024; WIdO 2022). Although the data covers around 85% of total antibacterial consumption (Bätzing-Feigenbaum et al. 2016), there is a small proportion of consumption that is not included. The limitations in data may result in aspects not being identified and addressed.

The selection of the years analysed also impacts the conclusions drawn. Different time periods may yield varying results, and the significance of the findings is influenced by the number of data points available. Since data were not available for all antibacterial substances across all years, this might introduce uncertainties in comparisons. The limited number of examined antibacterial substances and the factors considered may also affect the study’s outcomes.

The study employed specific criteria for statistical methods, including the choice of statistical procedures and the thresholds for significant correlations at two-sided significance levels of 0.01 and 0.05. A strong correlation coefficient was determined as above (+/−) 0.8. Altering these parameters could lead to different conclusions.

## Conclusions and further perspectives

Antibacterial drugs with overlapping indications often influence each other and show similarities in their consumption trends and changes (Figs. 7 and 8). For example, drugs with overlapping indications, e.g. for respiratory tract infections, show consumption trajectories that are related to each other, which is also reflected in significantly strong correlations (Tables 3, 4 and 5). In contrast, niche substances with specific therapeutic applications tend to be more independent from changes in consumption of other antibacterial drugs. Their limited treatment spectrum and lack of overlap with broader therapeutic areas lead to distinct and stable patterns of use (Tables 5 and 6).

These findings highlight a fundamental distinction between antibacterial drugs: those being stronger influenced by interdependencies and external factors, which often show fluctuating trends, and those with more independent patterns and stable trajectories. Although causality cannot be definitively established, the observed patterns of consumption suggest that antibacterial drugs share similarities and influence each other within their consumption trends (Figs. 7 and 8). Trends and changes in consumption can often be attributed to identifiable influences such as direct interactions between antibacterial drugs, including competition and substitution, or external factors such as changes in guidelines, costs, bacterial resistance rates or regulatory restrictions. Frequently, a change in the consumption of one drug leads to a corresponding shift in another. However, a significant correlation does not imply causality, as drugs may be influenced in a similar way due to external influences, such as the decline within the COVID pandemic, and therefore appear correlated without exerting direct influence on one another. On the other hand, certain dependencies are well-documented and plausible, such as changes in guidelines or DDD costs, which lead to increased use of the recommended or more affordable drug and a corresponding decline in alternatives.

Understanding the interdependencies of antibacterial drug consumption is essential for assessing how changes in the use of one drug affect others. A better understanding of consumption trends enhances the ability to anticipate broader shifts in antibacterial consumption, particularly when there are substantial changes in the use of specific drugs. Such insights can improve the accuracy of predictive models (Bindel and Seifert 2025) and guide strategies for rational prescribing and antimicrobial stewardship. Prototypical consumption trajectories and characteristics were identified among the 15 analysed drugs, differentiating them based on peak popularity, volatility over time and its overall consumption trend.

There is a suggestion that popular antibacterial drugs, expressed by their high consumption volume and widespread use, have a stronger influence on the development of other drugs than less commonly prescribed drugs. This influence is observed as a trend rather than a universal pattern (Fig. 8) and cannot be fully substantiated by data. In particular, the most commonly prescribed drugs show relatively stable levels of significant correlations, while less commonly prescribed drugs show greater variability (Table S1). Factors such as DDD costs, treatment indications and overlapping therapeutic contexts could be indirect influences which are driving this dynamic (Bindel and Seifert 2024a, 2024b), with the consumption volume being the outcome rather than the introducing factor. Changes in the use of these widely prescribed drugs often trigger significant shifts in other drugs, either through substitution or redistribution of consumption.

The relationship between antibacterial drug consumption and its influencing factors is highly complex. However, potential connections between these drugs can be identified through the significance and direction of correlations in their consumption volumes (Fig. 8). These relationships can help predict broader antibacterial consumption patterns and highlight the role of widely used drugs in influencing prescribing trends.

Future research should extend the analysis to additional antibacterial drugs, other European countries and alternative classes of agents to validate and generalise these findings. In addition, further research for other influencing factors, such as the long-term effects of the COVID pandemic, is essential to preserve the efficacy of antibacterial drugs and improve predictive approaches. These efforts will support evidence-based prescribing and strategies for effective antibacterial stewardship.

## Supplementary Information

Below is the link to the electronic supplementary material.

Supplementary file1 (DOCX 14.3 MB)

## Data Availability

All source data for this study are available upon reasonable request from the authors.

## Abbreviations

AMR: Antimicrobial resistance
AVR: Arzneiverordnungsreport (Drug Prescription Report regarding the statuary health system of Germany, includes outpatient prescriptions defined daily dose being dispensed from pharmacies)
DDD: Defined daily dose
ENT: Upper respiratory tract infections
GKV: Gesetzliche Krankenversicherung (statutory health insurance)
RKI: Robert Koch Institute
SPSS: Statistical Product and Service Solutions (software for statistical analyses, IBM)
TOP15: Group of the 15 most prescribed antibacterial drugs (based on the year 2022)
UTI: Urinary tract infections
