# Supplementary material for: Similarities in the consumption trajectory of antibacterial drugs in the outpatient care sector in Germany from 1986 to 2022: identification of shared patterns, correlation analysis of prescribed defined daily dose and assessment of underlying influences
Source: Naunyn Schmiedebergs Arch Pharmacol. 2025 Apr 23;398(10):14143–64. doi: 10.1007/s00210-025-04165-0 (PMC12511235; doi:10.1007/s00210-025-04165-0)

**Similarities in the consumption trajectory of antibacterial drugs in the outpatient care sector in Germany from 1986 to 2022: Identification of shared patterns, correlation analysis of prescribed Defined Daily Dose and assessment of underlying influences**

**Lilly Josephine Bindel and Roland Seifert**

Supplemental Tables

***Table S1:*** *Characteristics for the correlation analysis of prescribed DDD. Antibacterial drugs are ranked according to their consumption volume in 2022 (Ludwig et al. 2024). Dependency is assessed on the basis of the number of significant correlations: substances with more than eight significant correlations are classified as ‘dependent’, meaning that their trajectory is influenced by other antibacterial drugs and overall consumption trends. Those with an exceptionally low number of correlations, less than eight, are categorized as ‘independent’, meaning that their trajectory does not share many significant similarities with other antibacterial drugs and are not trat strongly influenced by overall consumption trends*

| **Ranking** | **Antibacterial substance** | **Number of significant correlations (max. 14)** | **Number of strong significant correlations** | **Number of significant positive correlations** | **Number of significant negative correlations** | **Number of strong positive correlations** | **Number of strong negative correlations** | **Assessment of dependency** |
| --- | --- | --- | --- | --- | --- | --- | --- | --- |
| 1 | Amoxicillin | 10 | 1 | 7 | 3 | 1 | 0 | dependent |
| 2 | Cefuroxime axetil | 13 | 4 | 8 | 5 | 2 | 2 | dependent |
| 3 | Doxycycline | 11 | 3 | 3 | 8 | 2 | 1 | dependent |
| 4 | Amoxicillin clavulanic acid | 11 | 3 | 7 | 4 | 2 | 1 | dependent |
| 5 | Clindamycin | 12 | 2 | 8 | 4 | 1 | 1 | dependent |
| 6 | Azithromycin | 12 | 2 | 7 | 4 | 1 | 1 | dependent |
| 7 | Phenoxymethylpenicillin | 12 | 3 | 3 | 9 | 2 | 1 | dependent |
| 8 | Sulfamethoxazole-Trimethoprim | 11 | 5 | 3 | 8 | 2 | 3 | dependent |
| 9 | Nitrofurantoin | 3 | 0 | 2 | 1 | 0 | 0 | independent |
| 10 | Ciprofloxacin | 8 | 1 | 6 | 2 | 1 | 0 | dependent |
| 11 | Clarithromycin | 4 | 1 | 3 | 1 | 0 | 1 | independent |
| 12 | Cefaclor | 10 | 2 | 7 | 3 | 2 | 0 | dependent |
| 13 | Cefpodoxime | 8 | 2 | 5 | 3 | 2 | 0 | dependent |
| 14 | Pivmecillinam | 7 | 7 | 2 | 5 | 2 | 5 | independent |
| 15 | Roxithromycin | 9 | 1 | 3 | 6 | 0 | 1 | dependent |

***Table S2:*** *Characterisation of consumption trajectory for each antibacterial drug. For each substance, the trend is categorized in fluctuating, stable, decreasing or increasing. The variability of consumption within a short period of time is considered fluctuating or stable. Furthermore, the development of recent years and the peak popularity is listed.*

| **Ranking** | **Antibacterial drug** | **Trend** | **Variability** | **Peak popularity** | **Recent development** | **Assessment of dependency** |
| --- | --- | --- | --- | --- | --- | --- |
| 1 | Amoxicillin | fluctuating | fluctuating | 2010s | fluctuating decline | dependent |
| 2 | Cefuroxime axetil | fluctuating | fluctuating | 2010s | fluctuating decline | dependent |
| 3 | Doxycycline | decreasing | stable | 1990s | gradual decline | dependent |
| 4 | Amoxicillin clavulanic acid | increasing | stable | 2020s | sharp increase | dependent |
| 5 | Clindamycin | decreasing | stable | 2000s | gradual decline | dependent |
| 6 | Azithromycin | increasing | fluctuating | 2010s | gradual increase | dependent |
| 7 | Phenoxymethylpenicillin | decreasing | stable | 1990s | gradual decline | dependent |
| 8 | Sulfamethoxazole-Trimethoprim | decreasing | stable | 1990s | gradual decline | dependent |
| 9 | Nitrofurantoin | increasing | fluctuating | 2010s | fluctuating increase | independent |
| 10 | Ciprofloxacin | decreasing | fluctuating | 2010s | gradual decline | dependent |
| 11 | Clarithromycin | decreasing | fluctuating | 2010s | sharp decline | independent |
| 12 | Cefaclor | decreasing | fluctuating | 2010s | consistent decline | dependent |
| 13 | Cefpodoxime | increasing | stable | 2020s | consistent increase | dependent |
| 14 | Pivmecillinam | increasing | stable | 2020s | sharp increase | independent |
| 15 | Roxithromycin | decreasing | fluctuating | 2000s | sharp decline | dependent |

***Table S3:*** *Overview of number of matching similarities for consumption trajectories, based on Table 2. If three characteristics match, the cell is orange-coloured. If two characteristics match, the cell is yellow-coloured****.*** *This table provides an overview for each compared pair how similar the consumption trajectory is. The darker coloured the respective cell, the more similar the consumption did develop.*

|  | **1** | **2** | **3** | **4** | **5** | **6** | **7** | **8** | **9** | **10** | **11** | **12** | **13** | **14** | **15** |
| --- | --- | --- | --- | --- | --- | --- | --- | --- | --- | --- | --- | --- | --- | --- | --- |
| **1 Amoxicillin** | - |  |  |  |  |  |  |  |  |  |  |  |  |  |  |
| **2 Cefuroxime axetil** | 3 | - |  |  |  |  |  |  |  |  |  |  |  |  |  |
| **3 Doxycycline** | 0 | 0 | - |  |  |  |  |  |  |  |  |  |  |  |  |
| **4 Amoxicillin clavulanic acid** | 0 | 0 | 1 | - |  |  |  |  |  |  |  |  |  |  |  |
| **5 Clindamycin** | 0 | 0 | 2 | 1 | - |  |  |  |  |  |  |  |  |  |  |
| **6 Azithromycin** | 2 | 2 | 0 | 1 | 0 | - |  |  |  |  |  |  |  |  |  |
| **7 Phenoxymethyl-pencillin** | 0 | 0 | 3 | 1 | 2 | 0 | - |  |  |  |  |  |  |  |  |
| **8 Sulfamethoxazole-Trimethoprim** | 0 | 0 | 3 | 1 | 2 | 0 | 3 | - |  |  |  |  |  |  |  |
| **9 Nitrofurantoin** | 2 | 2 | 0 | 1 |  | 2 | 0 | 0 | - |  |  |  |  |  |  |
| **10 Ciprofloxacin** | 2 | 2 | 1 | 0 | 1 | 1 | 1 | 1 | 2 | - |  |  |  |  |  |
| **11 Clarithromycin** | 2 | 2 | 1 | 0 | 1 | 1 | 1 | 1 | 2 | 3 | - |  |  |  |  |
| **12 Cefaclor** | 2 | 2 | 1 | 0 | 1 | 1 | 1 | 1 | 2 | 3 | 3 | - |  |  |  |
| **13 Cefpodoxime** | 0 | 0 | 1 | 3 | 1 | 1 | 1 | 1 | 1 | 0 | 0 | 0 | - |  |  |
| **14 Pivmecillinam** | 1 | 1 | 1 | 3 | 2 | 1 | 1 | 1 | 1 | 0 | 0 | 0 | 3 | - |  |
| **15 Roxithromycin** | 0 | 0 | 1 | 0 | 1 | 1 | 1 | 1 | 1 | 2 | 2 | 2 | 0 | 0 | - |

Supplemental Figures

***Fig. S1****: Correlation matrix of significant correlations for the antibacterial substance amoxicillin. Significant strong positive correlations have an increasing trend line, while negative correlations depict a decreasing trend line. The prescribed DDD of the reference substance are plotted on the X-axis and the prescribed DDD of the compared substance on the Y-axis. Correlations considered as strong are underlined.*

***
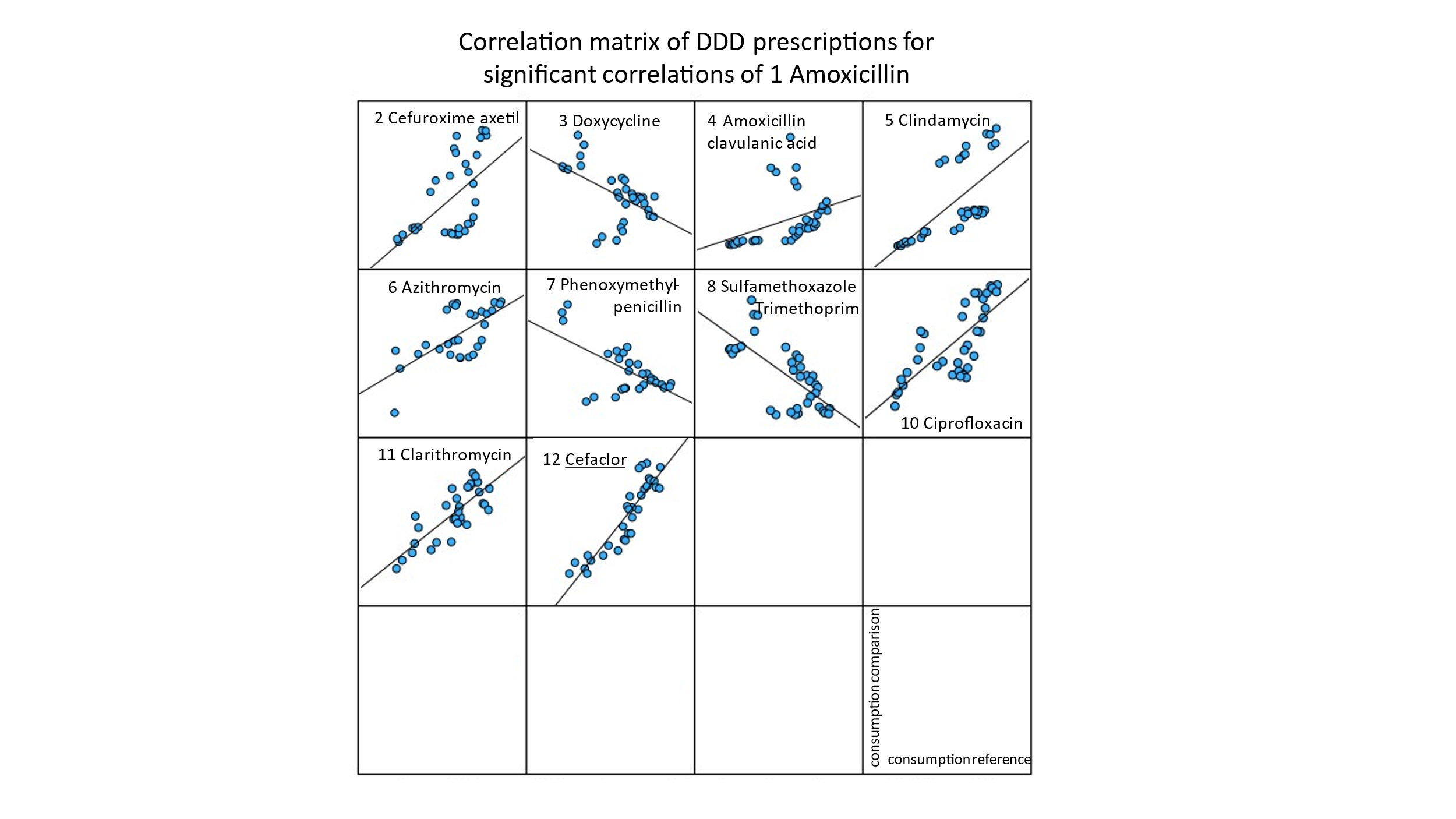
***

***Fig. S2:*** *Correlation matrix of significant correlations for the antibacterial substance cefuroxime axetil. Significant strong positive correlations have an increasing trend line, while negative correlations depict a decreasing trend line. The prescribed DDD of the reference substance are plotted on the X-axis and the prescribed DDD of the compared substance on the Y-axis. Correlations considered as strong are underlined.*

***
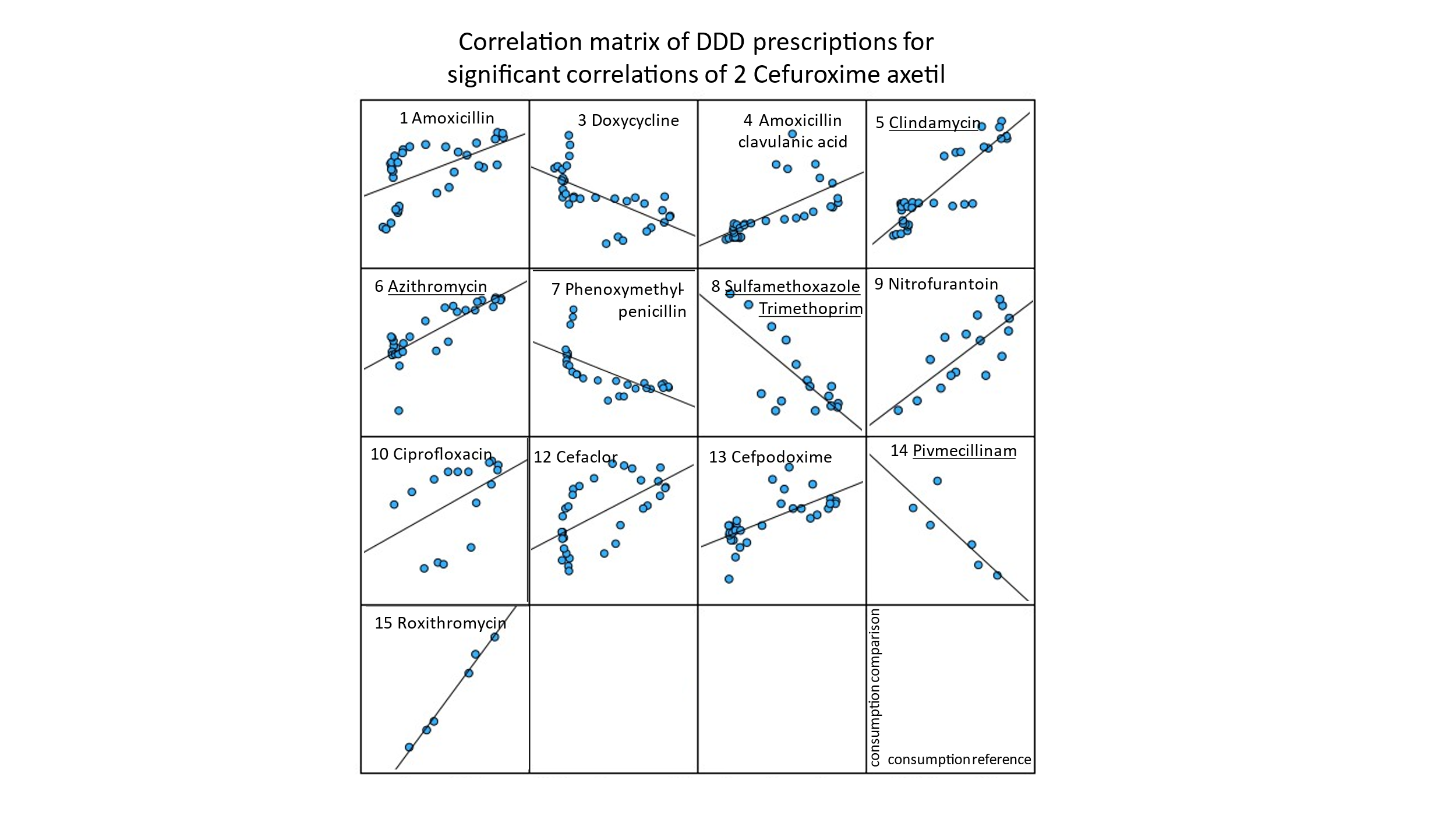
***

***Fig. S3:*** *Correlation matrix of significant correlations for the antibacterial substance doxycycline. Significant strong positive correlations have an increasing trend line, while negative correlations depict a decreasing trend line. The prescribed DDD of the reference substance are plotted on the X-axis and the prescribed DDD of the compared substance on the Y-axis. Correlations considered as strong are underlined.*

***
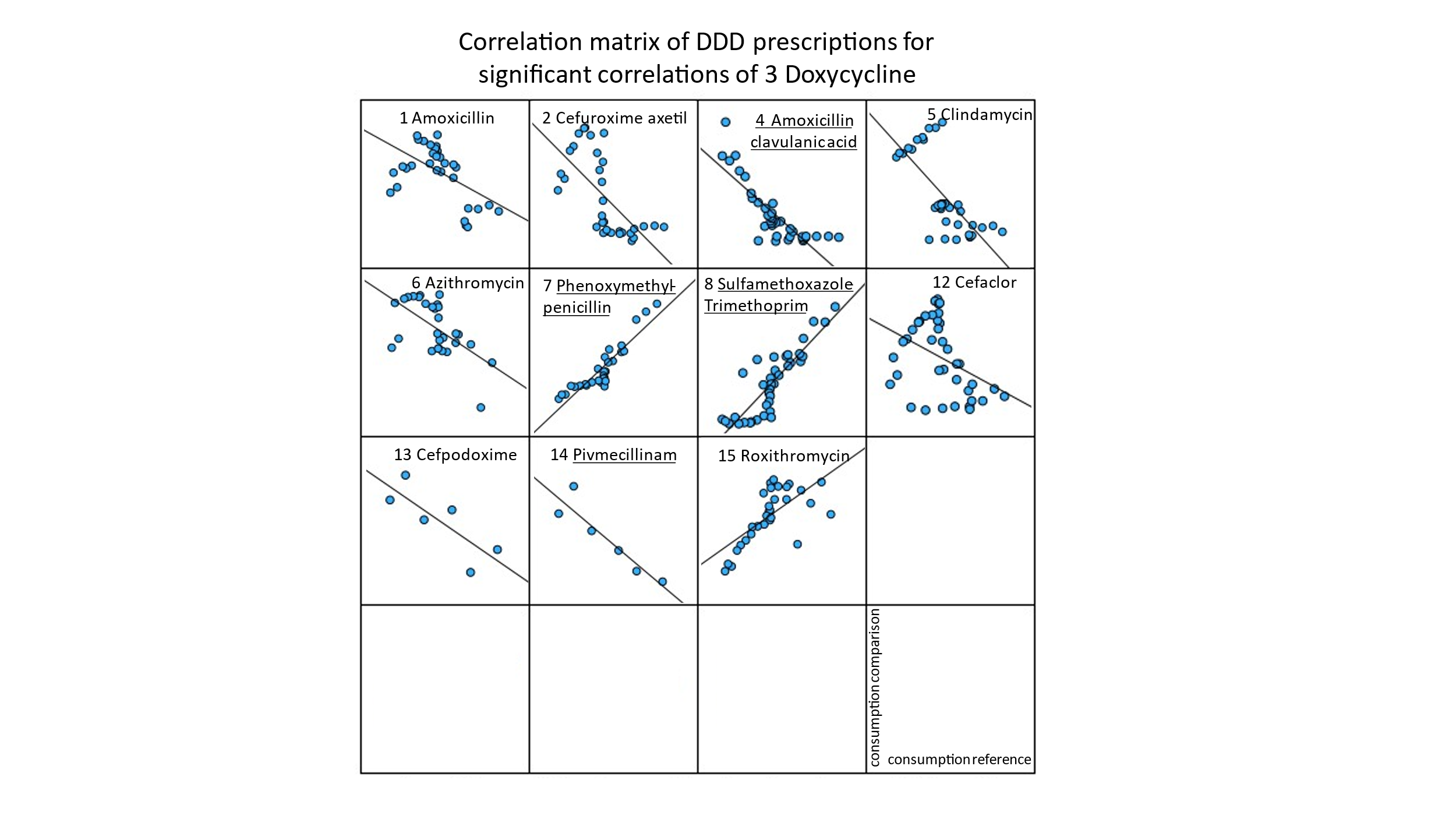
***

***Fig. S4:*** *Correlation matrix of significant correlations for the antibacterial substance amoxicillin clavulanic acid. Significant strong positive correlations have an increasing trend line, while negative correlations depict a decreasing trend line. The prescribed DDD of the reference substance are plotted on the X-axis and the prescribed DDD of the compared substance on the Y-axis. Correlations considered as strong are underlined.*

***
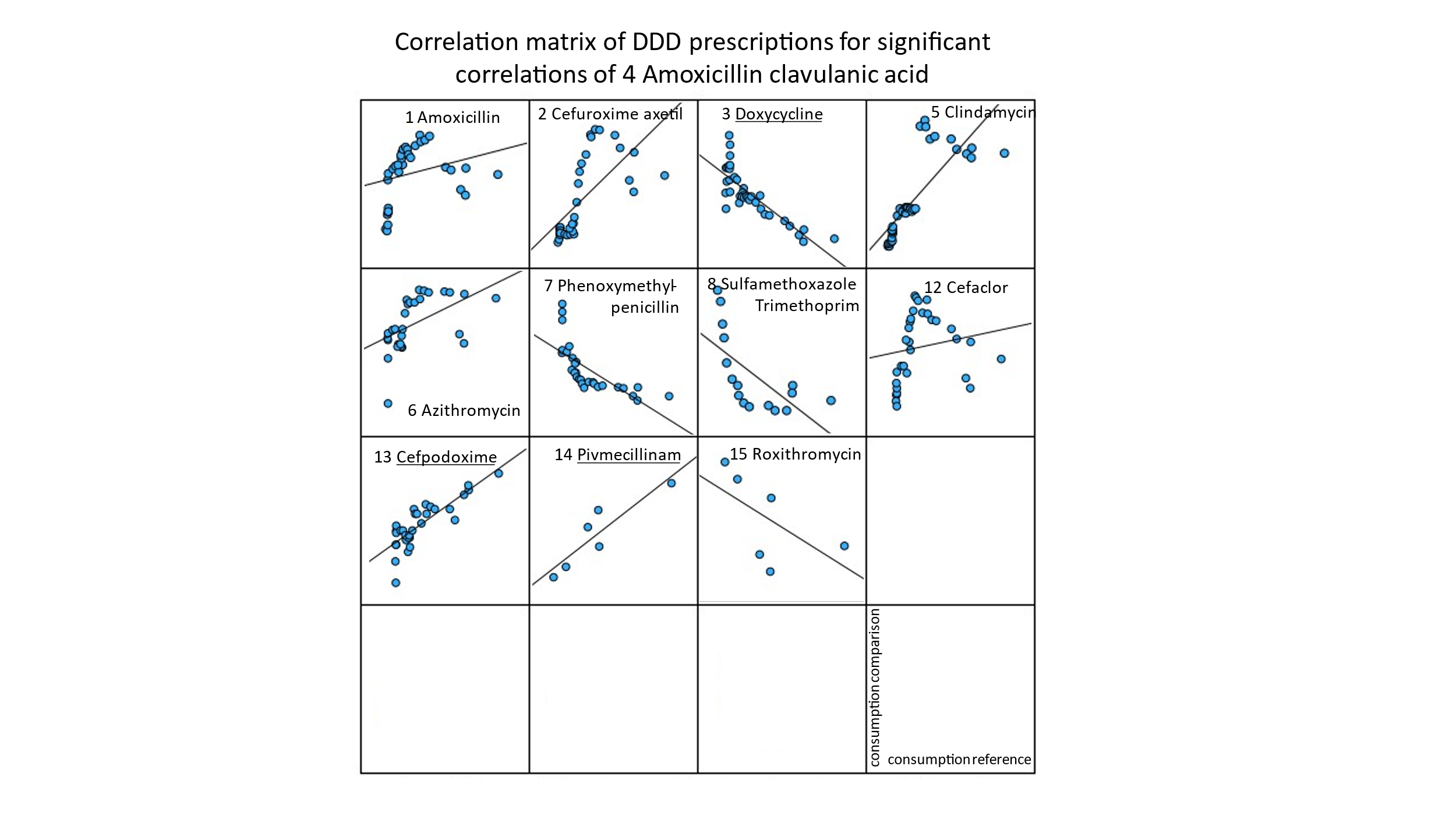
***

***Fig. S5****: Correlation matrix of significant correlations for the antibacterial substance clindamycin. Significant strong positive correlations have an increasing trend line, while negative correlations depict a decreasing trend line. The DDD prescriptions of the reference substance are plotted on the X-axis and the DDD prescriptions of the compared substance on the Y-axis. Correlations considered as strong are underlined.*


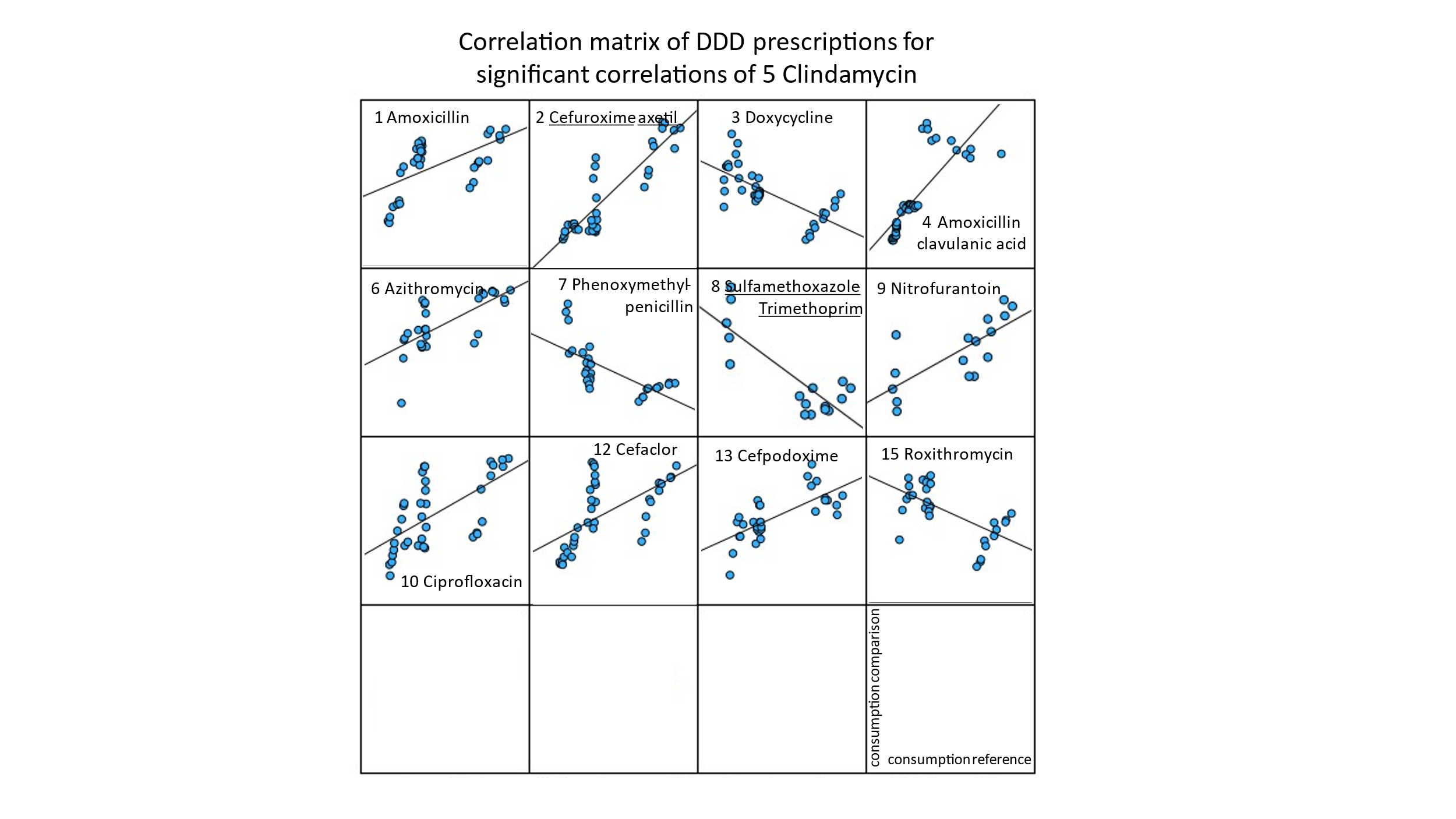


***Fig. S6****: Correlation matrix of significant correlations for the antibacterial substance azithromycin. Significant strong positive correlations have an increasing trend line, while negative correlations depict a decreasing trend line. The DDD prescriptions of the reference substance are plotted on the X-axis and the DDD prescriptions of the compared substance on the Y-axis. Correlations considered as strong are underlined.*


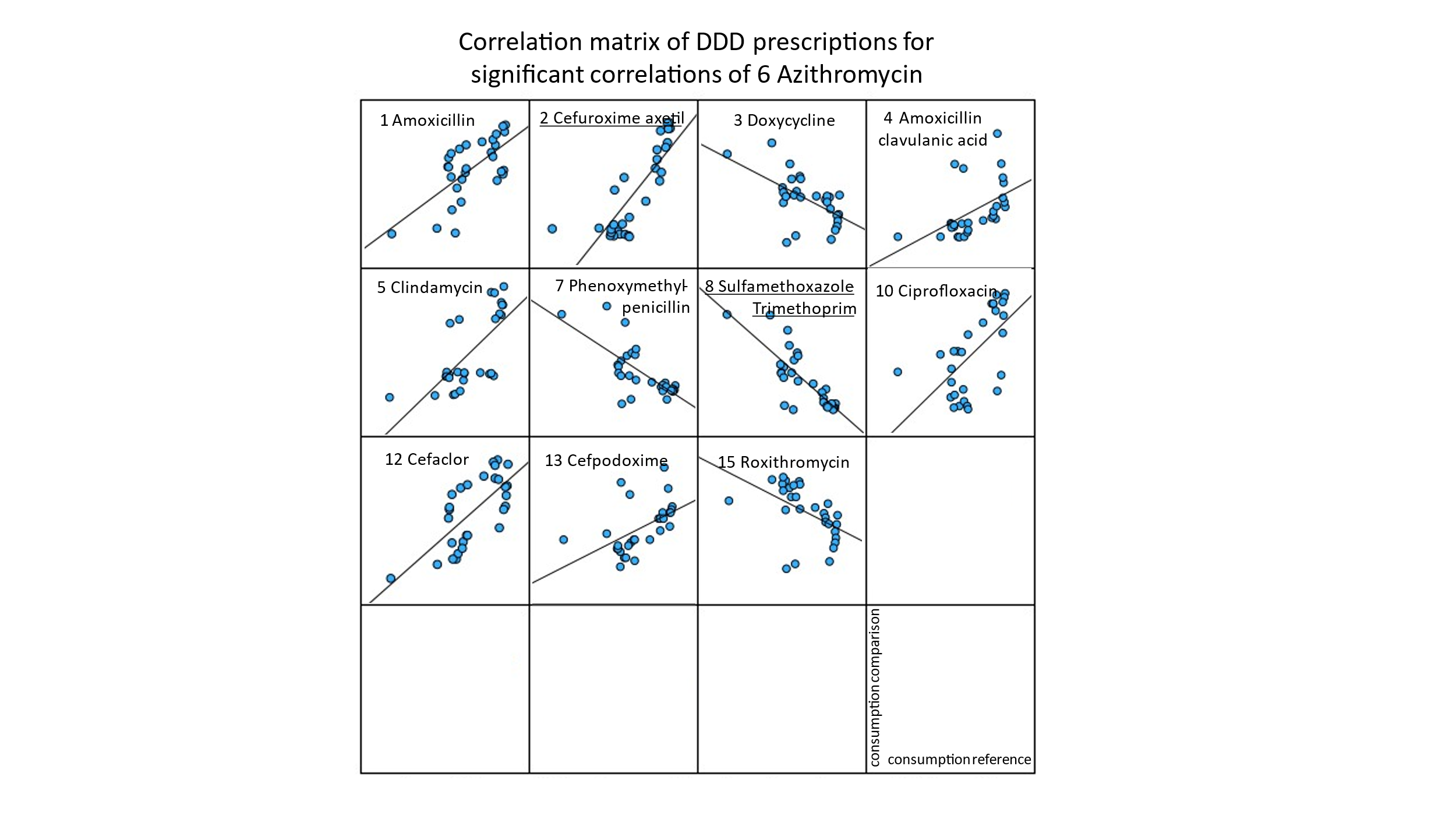


***Fig. S7****: Correlation matrix of significant correlations for the antibacterial substance phenoxymethylpenicillin. Significant strong positive correlations have an increasing trend line, while negative correlations depict a decreasing trend line. The DDD prescriptions of the reference substance are plotted on the X-axis and the DDD prescriptions of the compared substance on the Y-axis. Correlations considered as strong are underlined.*


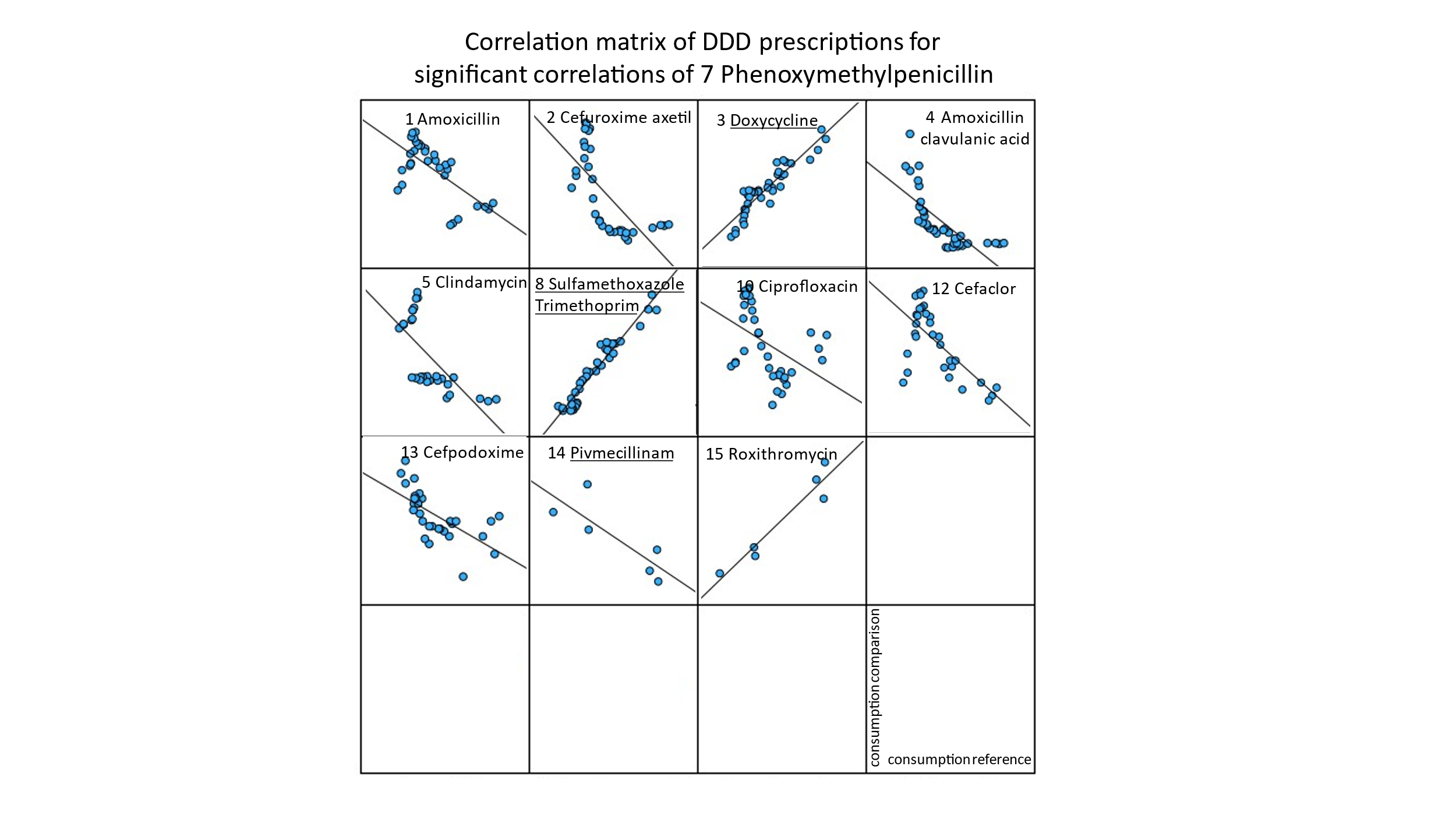


***Fig. S8****: Correlation matrix of significant correlations for the antibacterial substance sulfamethoxazole-trimethoprim. Significant strong positive correlations have an increasing trend line, while negative correlations depict a decreasing trend line. The DDD prescriptions of the reference substance are plotted on the X-axis and the DDD prescriptions of the compared substance on the Y-axis. Correlations considered as strong are underlined.*


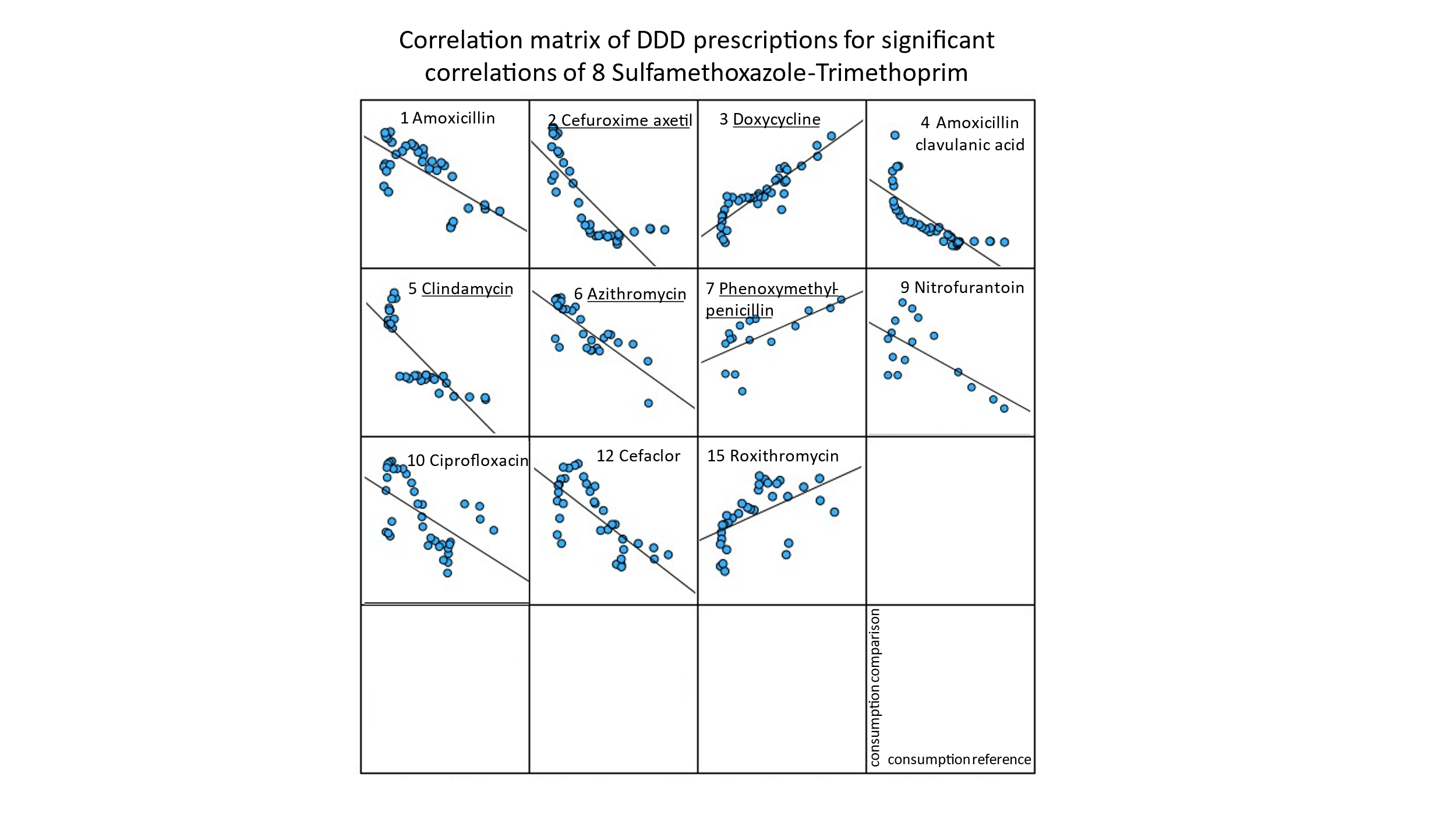


***Fig. S9****: Correlation matrix of significant correlations for the antibacterial substance nitrofurantoin. Significant strong positive correlations have an increasing trend line, while negative correlations depict a decreasing trend line. The DDD prescriptions of the reference substance are plotted on the X-axis and the DDD prescriptions of the compared substance on the Y-axis. Correlations considered as strong are underlined.*


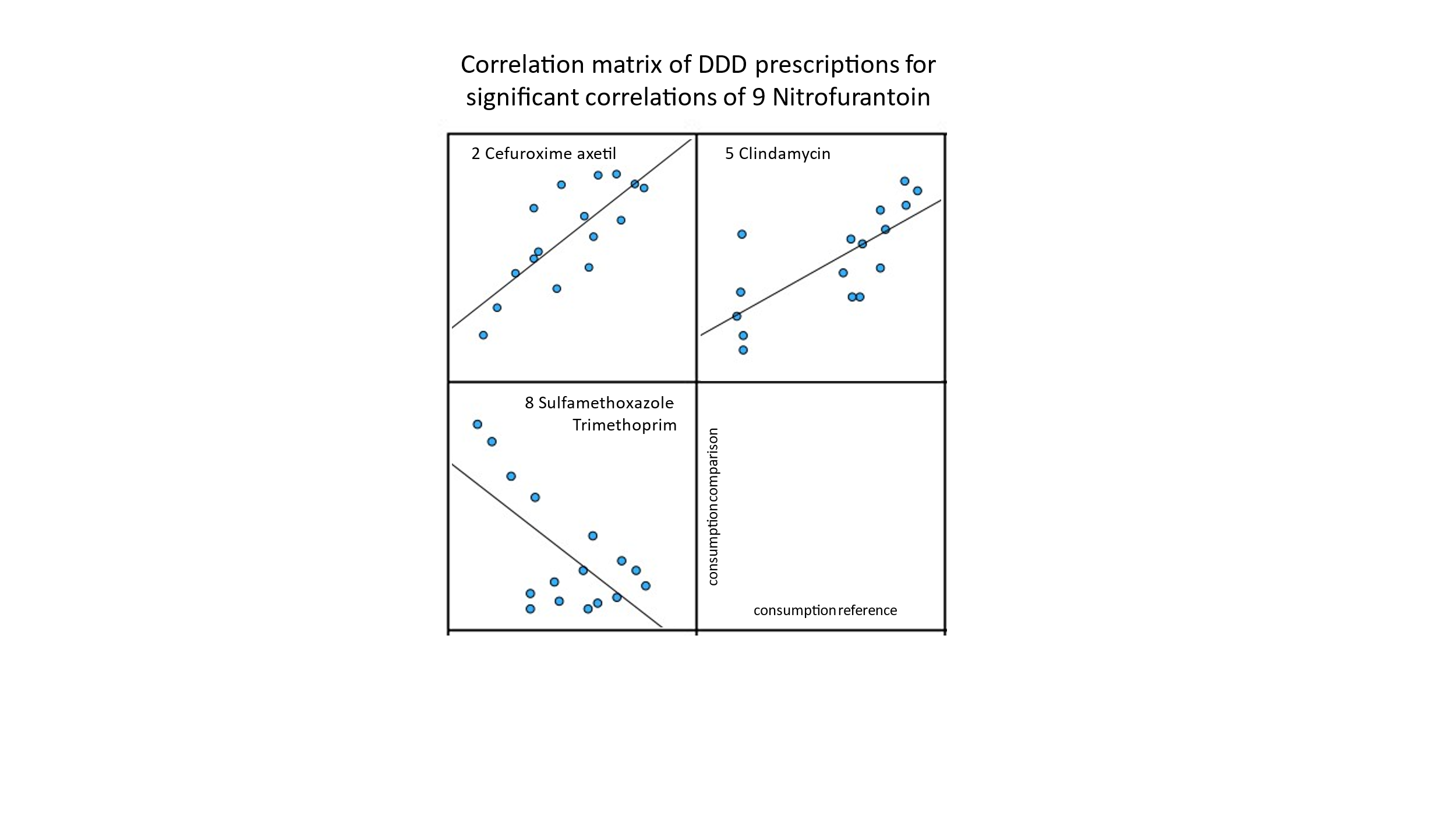


***Fig. S10****: Correlation matrix of significant correlations for the antibacterial substance ciprofloxacin. Significant strong positive correlations have an increasing trend line, while negative correlations depict a decreasing trend line. The DDD prescriptions of the reference substance are plotted on the X-axis and the DDD prescriptions of the compared substance on the Y-axis. Correlations considered as strong are underlined.*


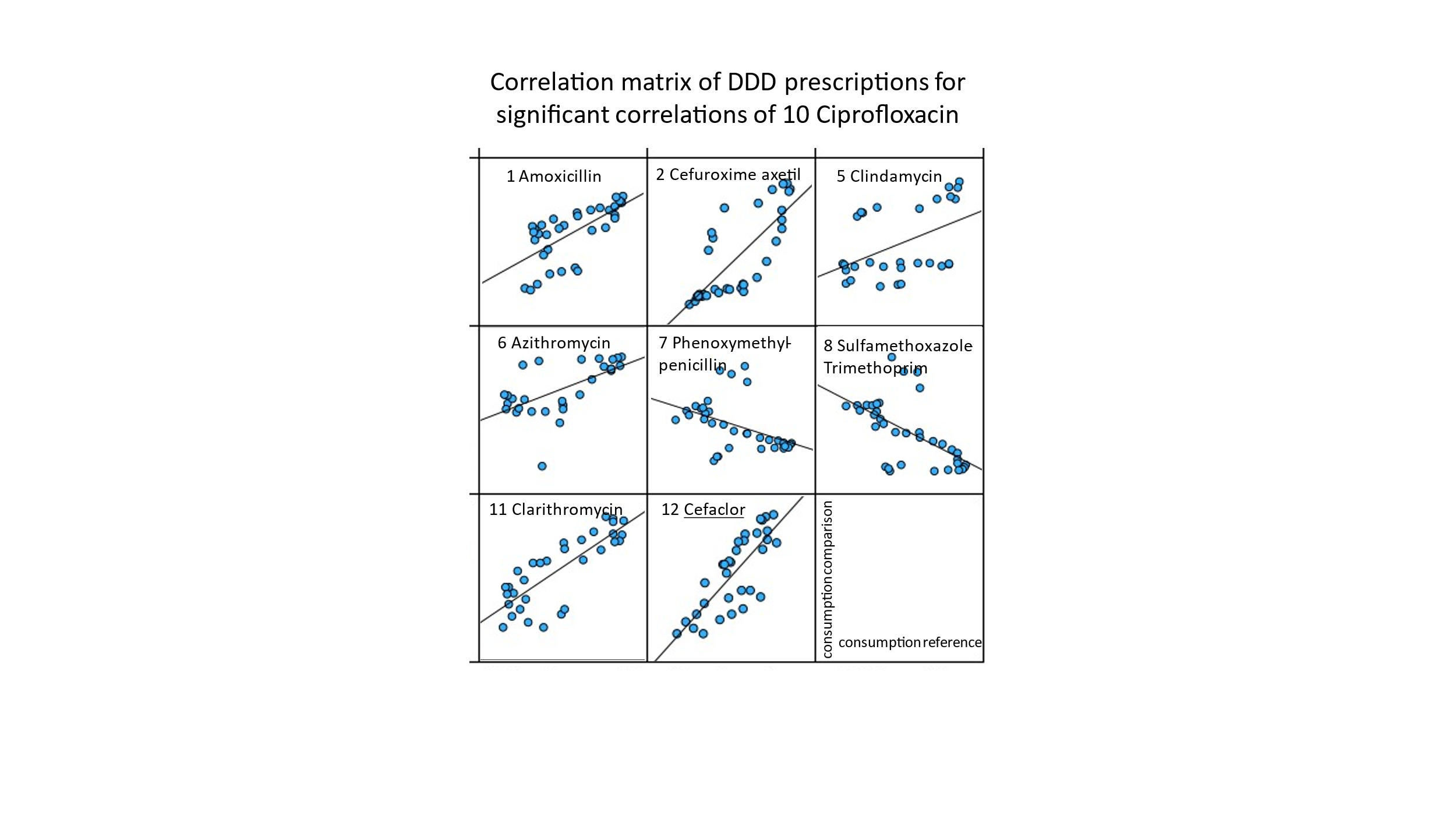


***Fig. S11****: Correlation matrix of significant correlations for the antibacterial substance clarithromycin. Significant strong positive correlations have an increasing trend line, while negative correlations depict a decreasing trend line. The DDD prescriptions of the reference substance are plotted on the X-axis and the DDD prescriptions of the compared substance on the Y-axis. Correlations considered as strong are underlined.*


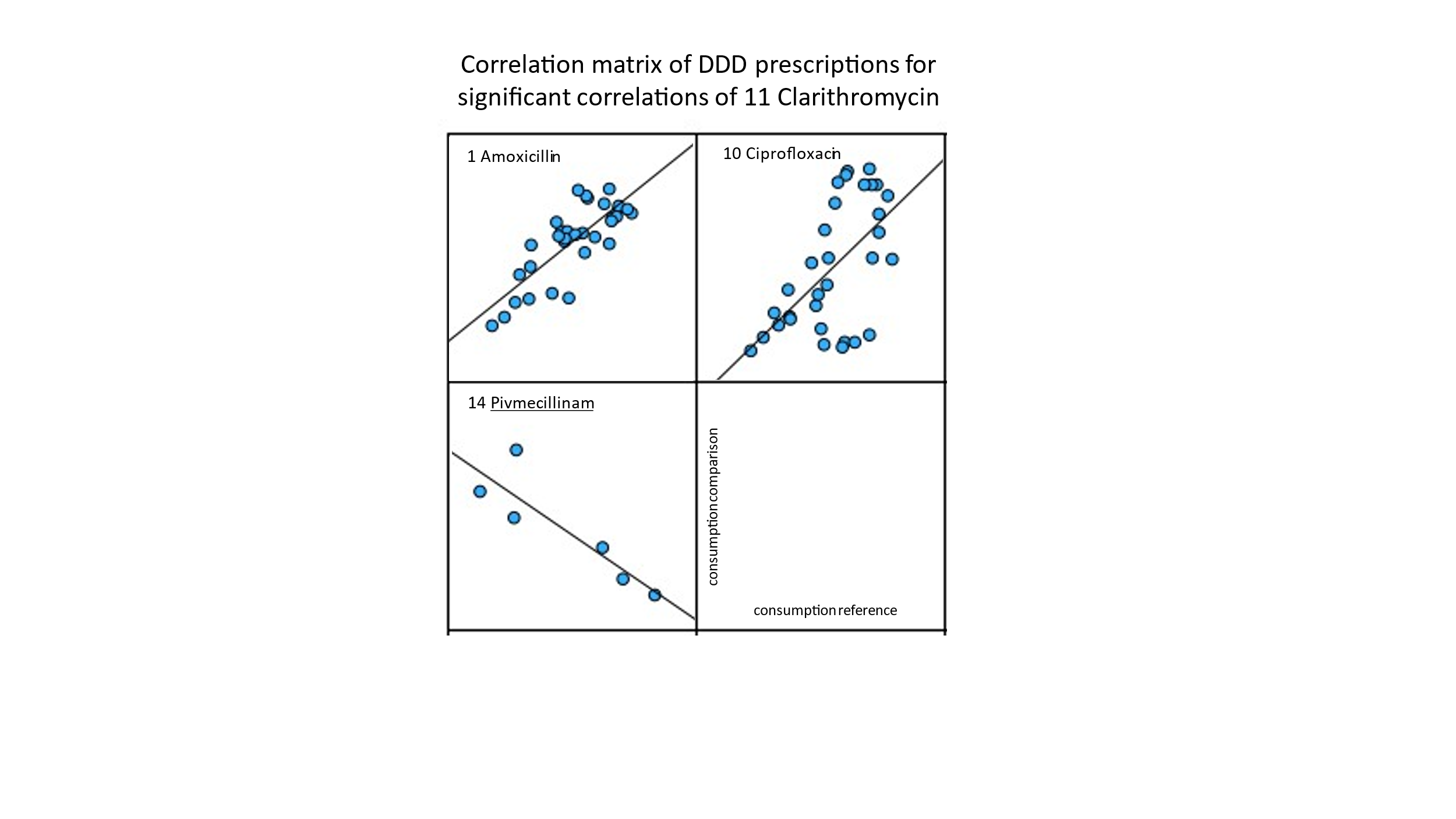


***Fig. S12****: Correlation matrix of significant correlations for the antibacterial substance cefaclor. Significant strong positive correlations have an increasing trend line, while negative correlations depict a decreasing trend line. The DDD prescriptions of the reference substance are plotted on the X-axis and the DDD prescriptions of the compared substance on the Y-axis. Correlations considered as strong are underlined.*


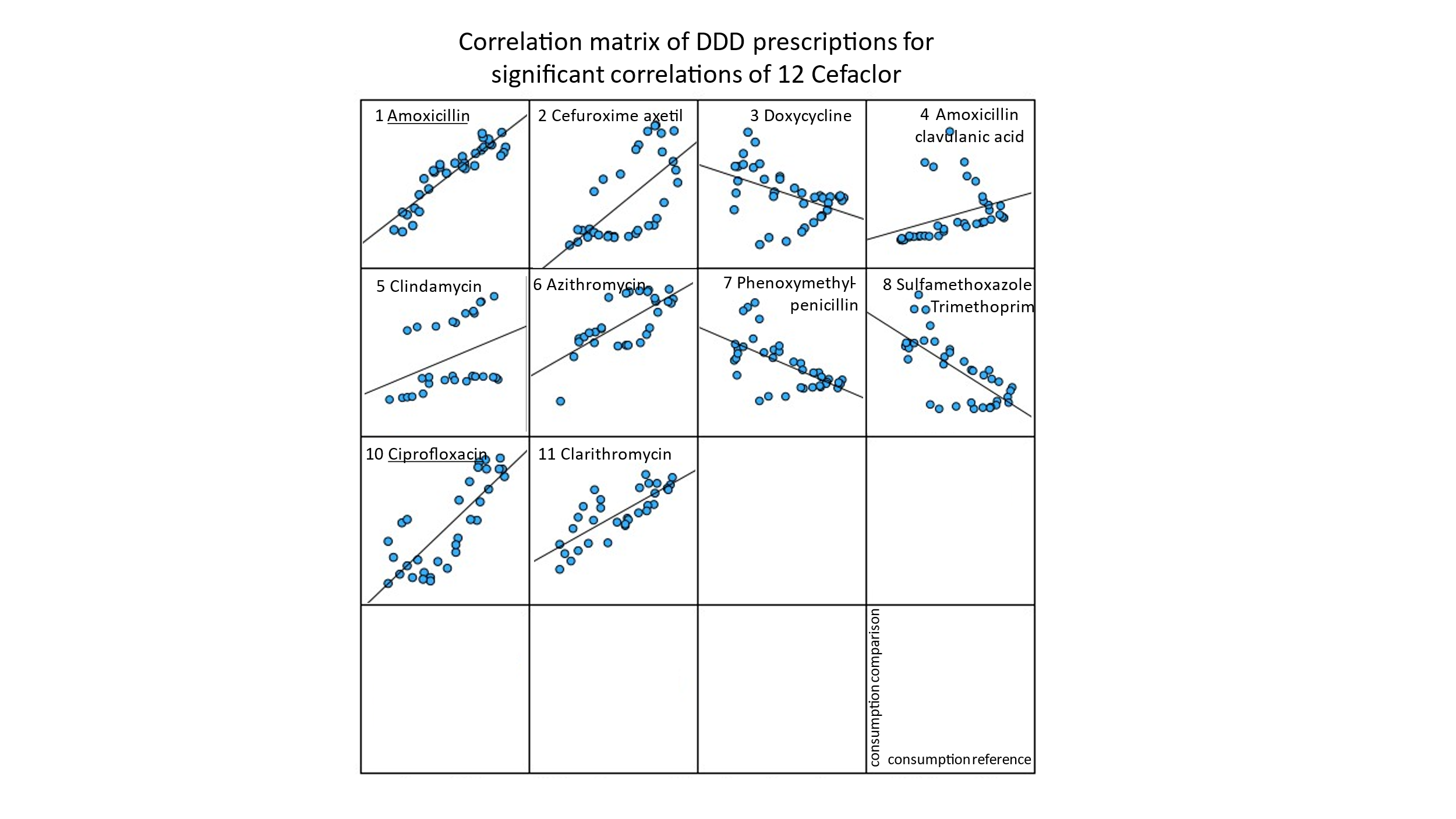


***Fig. S13****: Correlation matrix of significant correlations for the antibacterial substance cefpodoxime. Significant strong positive correlations have an increasing trend line, while negative correlations depict a decreasing trend line. The DDD prescriptions of the reference substance are plotted on the X-axis and the DDD prescriptions of the compared substance on the Y-axis. Correlations considered as strong are underlined.*


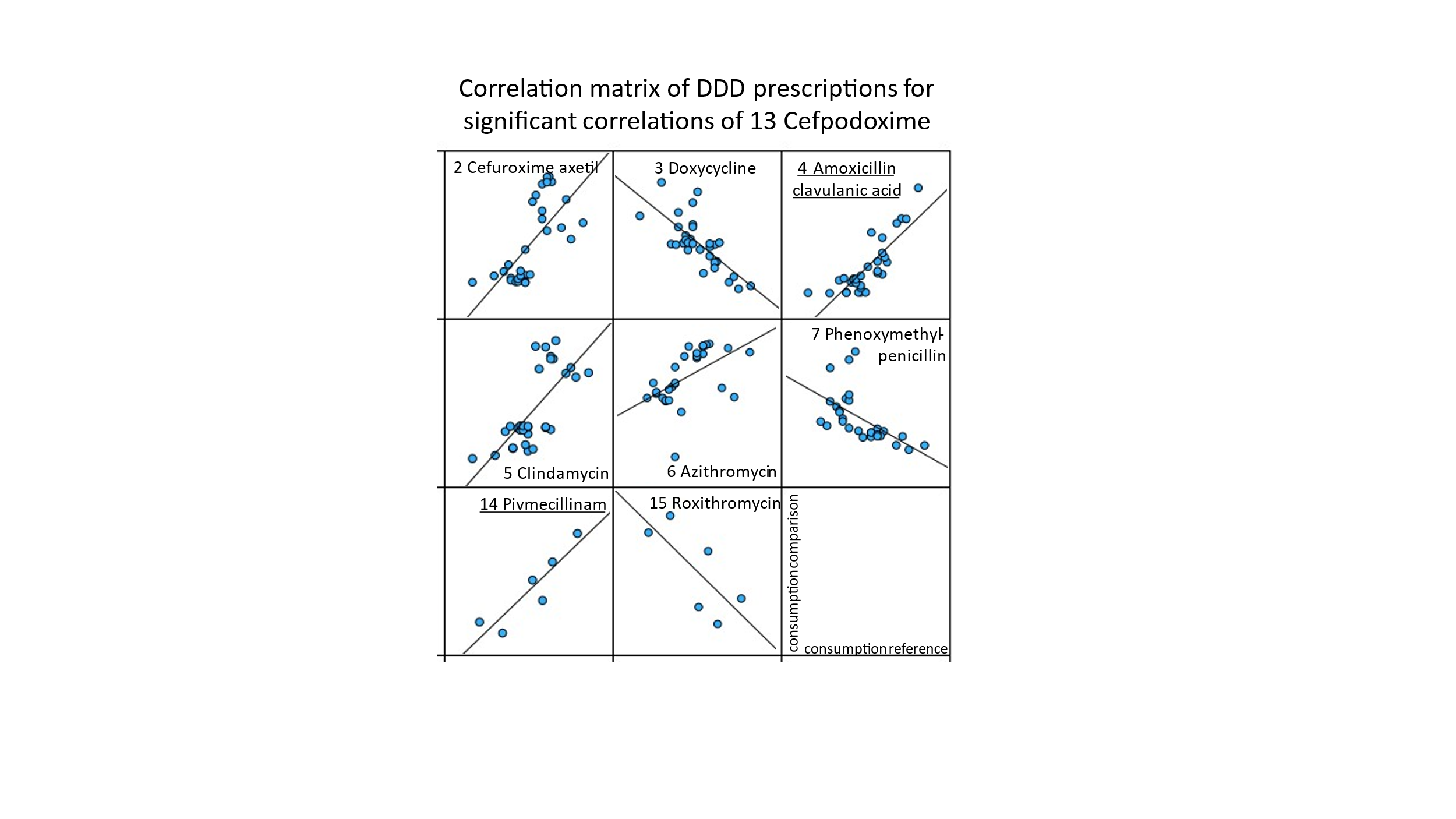


***Fig. S14****: Correlation matrix of significant correlations for the antibacterial substance pivmecillinam. Significant strong positive correlations have an increasing trend line, while negative correlations depict a decreasing trend line. The DDD prescriptions of the reference substance are plotted on the X-axis and the DDD prescriptions of the compared substance on the Y-axis. Correlations considered as strong are underlined.*


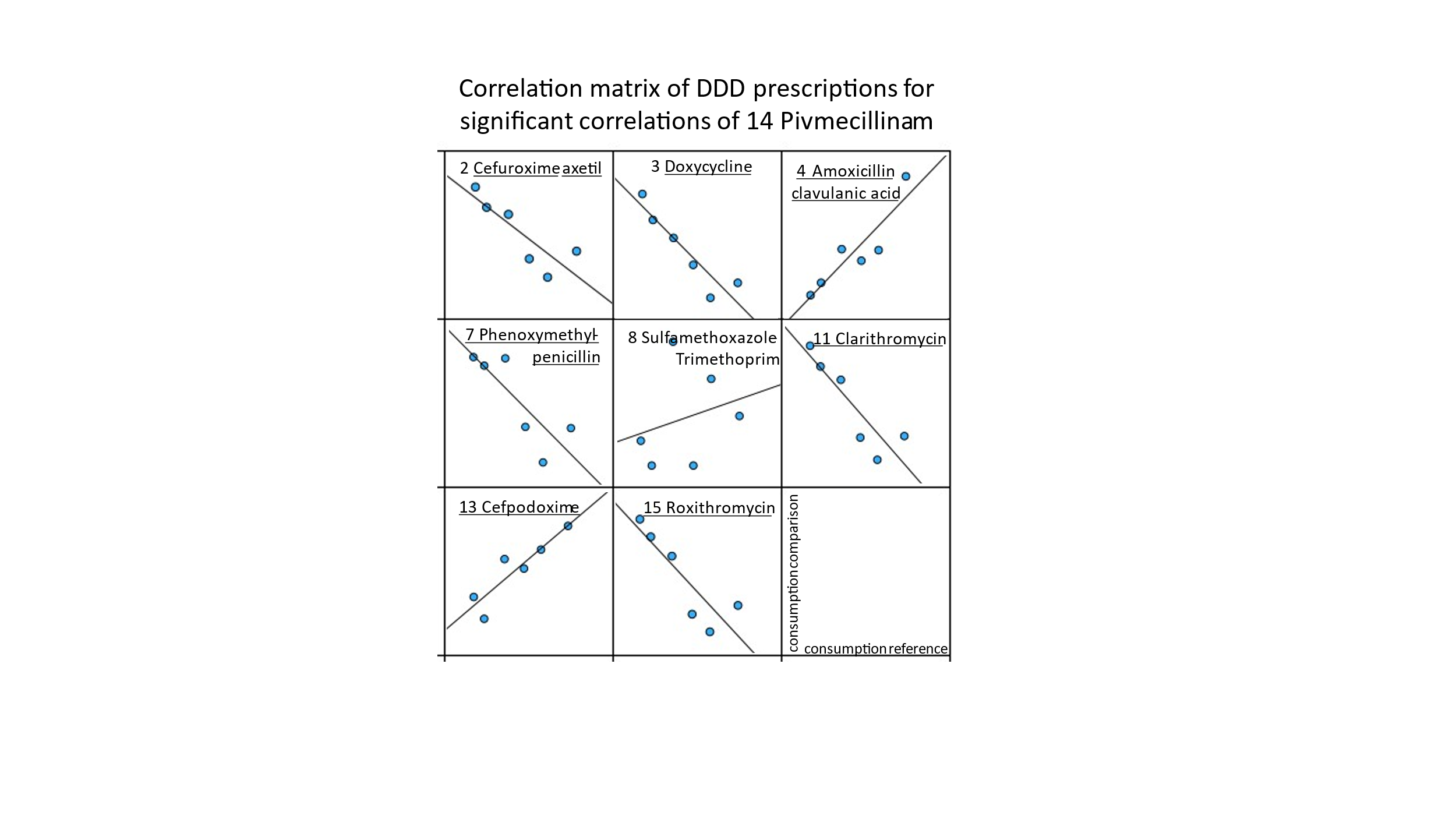


***Fig. S15****: Correlation matrix of significant correlations for the antibacterial substance roxithromycin. Significant strong positive correlations have an increasing trend line, while negative correlations depict a decreasing trend line. The DDD prescriptions of the reference substance are plotted on the X-axis and the DDD prescriptions of the compared substance on the Y-axis. Correlations considered as strong are underlined.*


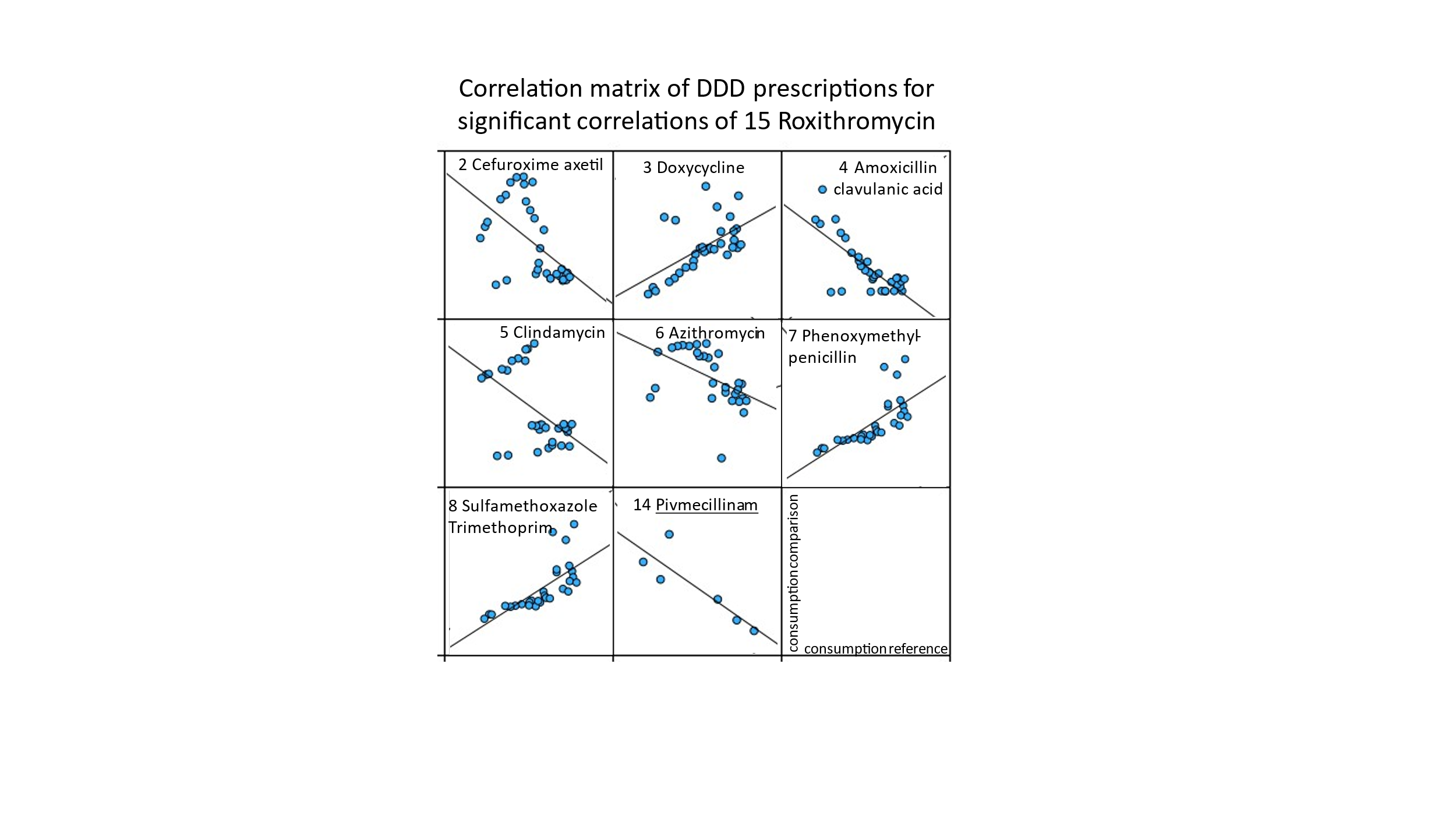

Supplement: Supplementary file 1 — Supplementary file1 (DOCX 14.3 MB) [file 210_2025_4165_MOESM1_ESM.docx]
